# Minimally Invasive Protocol for the Management of Unilateral Condylar Hyperplasia: Case Series on Seven Patients

**DOI:** 10.3390/jcm15072671

**Published:** 2026-04-01

**Authors:** Funda Goker, Daniele Hamaui, Giulia Tirelli, Aldo Bruno Gianni, Gianluca Martino Tartaglia, Sourav Panda, Massimo Del Fabbro, Diego Sergio Rossi

**Affiliations:** 1 Department of Biomedical, Surgical and Dental Sciences, University of Milan, 20122 Milan, Italy; danielehamaui@gmail.com (D.H.); giulia.tirelli@unimi.it (G.T.); gianluca.tartaglia@unimi.it (G.M.T.); 2 Fondazione IRCCS Ca’ Granda Ospedale Maggiore Policlinico, 20122 Milan, Italy; aldo.gianni@unimi.it (A.B.G.); diego.rossi@policlinico.mi.it (D.S.R.); 3 Department of Oral and Maxillofacial Surgery, Faculty of Dentistry, Istanbul Aydın University, 34295 Istanbul, Turkey; 4 Department of Periodontics and Oral Implantology, Institute of Dental Sciences, Siksha O Anusandhan University, Bhubaneswar 751030, India; drsaurav87@gmail.com

**Keywords:** intra-oral condylectomy, 3D printed titanium cutting guides, unilateral condylar hyperplasia, CAD/CAM, temporomandibular joint, mandibular asymmetry, orthognathic surgery

## Abstract

**Background/Objectives**: Unilateral condylar hyperplasia is an idiopathic condition that causes facial asymmetry and occlusal problems. Currently, traditional treatment protocol is the combination of orthognathic and extra-oral condylectomy surgery via pre-auricular incision, which can create aesthetic problems with additional risks of facial nerve damage. The purpose of this study was to report management of condylar hyperplasia patients through minimally invasive condylectomy that was planned via virtual methods. **Methods**: The custom-made cutting guides were produced, and unilateral condylectomy operations were performed via intra-oral approach. Orthognathic surgery with/without genioplasty operations were either done with condylectomy in one session or in an additional session. **Results**: Custom-made cutting guides produced by virtual methods provided easy operations without any need for additional extra-oral incisions. Planned osteotomies were transferred successfully from the virtual surgical plan and resections of the excess bone tissues were performed using novel piezo surgery devices. The bones were fixed to their pre-planned position using 3D-printed titanium plates. The patients healed without any complications. Results of this innovative virtually guided protocol tested showed functional and esthetic results without any extra-oral scars with no facial nerve damage. **Conclusions**: Combination of intra-oral condylectomy with orthognathic surgery using 3D-printed titanium cutting guides seems to be an advantageous approach with successful results in terms of aesthetics and function for management of mandibular condylar hyperplasia patients; however, there is an urgent need in the scientific literature for further clinical research with a larger number of subjects.

## 1. Introduction

Unilateral condylar hyperplasia (UCH) is a non-neoplastic condylar hyperactivity that causes facial asymmetry, lateral deviation, and consequently, occlusal, functional, and aesthetic problems [1,2,3,4,5]. In UCH patients, the increase in the vertical dimension on the affected side develops as the result of an idiopathic excessive unilateral growth of the mandibular condyle and is quite challenging for the maxillofacial surgeon [6]. The aetiology of UCH is still unclear; however, according to the literature, it can be seen following trauma or infection. Other possible causes are considered as genetic, hormonal, or embryogenic [1,6]. A classification of UCH was presented by Obwegeser and Makek in 1986, which divided UCH cases into two classifications: hemimandibular hyperplasia and hemimandibular elongation [7,8]. However, as mentioned in a more recent article, the patients can represent a blend of these two types [9].

UCH develops as result of osteoblastic hyperactivity and the patients are divided into two forms, active and inactive, which can be differentiated using Planar or SPECT (single photon emission computed tomography) methods [1]. UHC can be confirmed by these scintigraphy reports (for calculation of the one condylar uptake percentage versus condyle of the other side) for checking condylar hyperplasia-relative growth rates and to understand if the one of the condyles is still actively growing or if growth has ceased. The asymmetry resulting from the unilateral mandibular condylar hyperactivity is the result of the excessive unilateral growth of a mandibular condyle related to the contralateral. The differences of 10% or more between two condyles show hyperactivity on one side and defined as “active form” [1,7]. Currently, the treatment of UCH is managed by the combination of orthodontics and condylectomy surgeries with aesthetic and functional outcomes [3,10]. Various protocol alternatives were reported in the literature describing appropriate timing for orthodontics therapy as pre-surgical or post-surgical, with or without CAD/CAM methods and different surgical approaches, such as high condylectomy during the active phase or low condylectomy, orthognathic surgery, or combination of condylectomy with orthognathic surgery [6,11,12,13,14,15,16,17,18,19,20,21].

Today, modern technology offers alternatives to traditional surgery protocols such as intra-operative real-time navigation and CAD/CAM (computer-aided design and computer-aided manufacturing) methods that use DICOM data measurements and segmentations for manufacturing custom-made models, surgical guides and surgical plates [22,23]. Maxillofacial surgery demands precise skeletal repositioning to optimize both functional outcomes and facial aesthetics [22,23]. Several reports in the literature have compared fully guided, computer-assisted approaches and conventional free-hand techniques [22,23]. Currently, CAD/CAM techniques utilize 3D modeling and rapid prototyping to generate custom-made cutting guides, osteosynthesis plates, and splints specific for each case. As a result, a workflow is created that facilitates a precise pre-operative simulation, which is replicable and predictable for intraoperative resection, reconstruction and re-positioning. In brief, data from CT (computer tomography) scans is used to create a 3D model of the patients’ anatomy, virtual surgical planning is done using the 3D models to simulate position of the bones, osteotomies and patient-specific cutting guides and plates for reconstruction are designed and are manufactured using 3D printing methods. These custom-made models, guides and plates are used during surgery to obtain more accurate results than traditional surgery. On the other hand, conventional protocols just rely on classical cephalometric analyses, conventional radiographs and manual fabrication processes, which are mostly dependent on the surgeon’s experience, which is prone to inter- and intra-operator variability [22]. These recent advances in CAD/CAM technologies provide beneficial alternative protocols for management of several deformities in the maxillofacial region, including condylar hyperplasia cases [15,16,17,18,19].

Currently, traditional treatment protocol is the combination of orthognathic and extra-oral condylectomy surgery. However, extra-oral condylectomy operation via pre-auricular incision represents unesthetic results with additional risks of facial nerve damage. To overcome these complications, minimally invasive surgical protocol for intra-oral condylectomy was assessed in the literature using novel techniques that are based on CAD/CAM planning and surgical resection of the condylar process using customized three-dimensional (3D)-printed cutting guides [3,5,10]. Currently, the number of reports is still very limited. According to the knowledge of the authors of this report, there are just three clinical reports by two groups of authors with very small number of patients [3,5,10]. There is a lack of research in the literature, and, at this point, the aim of this pilot case series was to evaluate the outcomes of minimally invasive intra-oral condylectomy operation for the management of unilateral condylar hyperplasia for better aesthetic results and reduced risks of nerve damage. For this purpose, the surgeries were planned via virtual methods. The custom-made CAD/CAM cutting guides were positioned and unilateral condylectomy and orthognathic operations were performed via intra-oral approach using piezoelectric instruments.

## 2. Materials and Methods

This preliminary study represents management of seven unilateral condylar hyperplasia patients. All the patients were treated in Maxillo-facial Department at Ospedale Maggiore Policlinico of Milan, Italy. The rehabilitation of the patients included orthodontic therapy and surgical interventions (intra-oral condylectomy, orthognathic surgery with/without genioplasty) utilizing 3D-printed titanium cutting guides and plates using CAD/CAM methods (SINTAC, Trento, Italy) or Materialise (Leuven, Belgium). The study protocol followed the principles laid down in the Declaration of Helsinki on medical protocol and informed consent was obtained from the patients for the surgeries and for using data including photos for scientific publications. The study protocol was approved by the Ethics Committee of Milano Area B with date 10/01/2019 Act 2215/2018, Determinazione No: 0015.

Inclusion criteria: Skeletally mature, unilateral condylar hyperplasia patients with asymmetry of the lower third, who accepted to participate the study and signed consent forms, minimum mouth opening of 38 mm. UCH cases included in work belonged to the two classifications or a blend of these two types (both active and inactive forms) [7,9].

Exclusion criteria: Oncological conditions; treatment with anticoagulant medications (INR greater than 2); treatment with anticonvulsants; patient’s inability to maintain reasonable standards of home hygiene, in accordance with study requirements; patients with a history of drug or alcohol abuse; patients on chronic steroid therapy; psychosis; women who were pregnant or breastfeeding at the time of enrollment or surgery; lack of patient cooperation; other uncontrolled systemic conditions that preclude surgical procedures.

**Figure 1:**
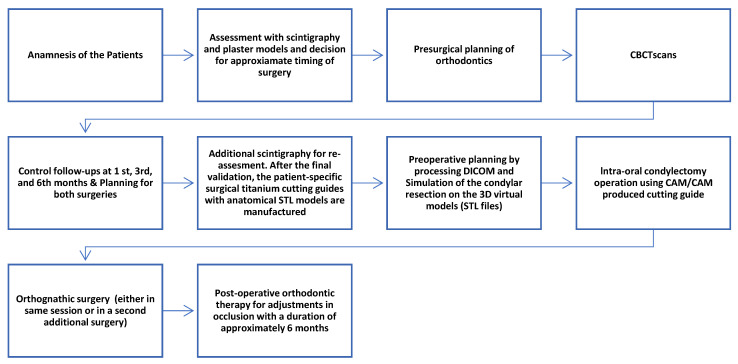
Flowchart of the protocol utilized in this study.

In Figure 1, the diagram for the steps of the treatment protocol used in this study can be seen as a brief explanation.

First visit—Anamnesis of the Patients

The study included seven Caucasian female patients (with a median age of 25.3, SD 4.4 years) referred for facial asymmetry of the lower third and skeletal dysmorphisms with hemi-mandibular lengthening because of active mandibular condylar hyperplasia.

There was a severe facial asymmetry in all the patients that created aesthetic and functional problems which were the main reason for the first visit. The subjects had normal height and weight with no systemic conditions or pathologies, or any psychiatric problems. There were no stories of any previous traumas, or surgical interventions/hospitalizations. General health status was ASA1 for each. None were under orthodontic treatment. The planned orthodontic and surgical procedures, the risks, the costs, the waiting times were explained to the patients and their parents.

Second visit (approximately after 3 months): Re-assessment with scintigraphy and plaster models. Wisdom teeth extractions and pre- and/or post-surgical orthodontics were scheduled.

Third visit (approximately at the following 6 months):

The patients were sent to a radiologist for additional scintigraphy report, for calculation of the left condylar uptake percentage versus right condyle or vice versa in order to check condylar hyperplasia-relative growth rates. Results showed more than 10% difference and surgeries were planned. The patients reported to have completed wisdom teeth extractions and pre-surgical orthodontic rehabilitations were also scheduled at this stage.

Pre-operative protocol:

The operations were planned as one side (right/left) proportional condylectomy for symmetrization via intra-oral access utilizing CAD/CAM custom-made technique under general anesthesia.

Pre-operative planning was done by processing DICOM (Digital Imaging and Communications in Medicine) files via web-based service with the support of a medical engineer from the company (SINTAC, Trento) or Materialise (Leuven, Belgium). Planning included resection which was simulated on the 3D virtual models (STL files: Standard Tessellation Language files used for stereolithography). Dimensions of left/right side mandible were matched with that of right TMJ by an osteotomy through cutting guide to remove excess osseous tissue. After the final validation, the patient-specific surgical titanium cutting guides with STL models were created within 10 working days. Pre-surgical orthodontics were applied using fixed braces. The pre-operative protocol included routine blood tests, ECG, chest x-ray, SARS-COV2 screening (by nasopharyngeal swab, which showed negative result) and anesthetic visit in pre-hospitalization (a day before the surgery). No contraindications were found to postpone or cancel the surgeries.

**Figure 2:**
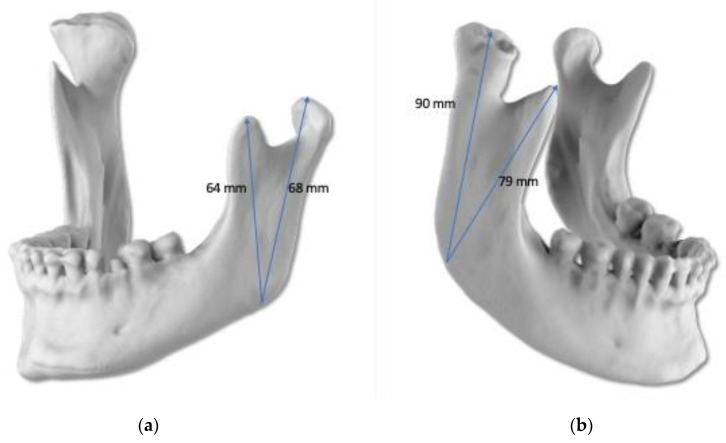
Pre-operative analyses and planning via virtual methods. Pre-operative measurements of the mandibular condyle for comparison of right and left sites. (**a**) The measurements of the left side mandibular condyle and coronoid; (**b**) measurements of the right-side mandibular condyle and coronoid.

**Figure 3:**
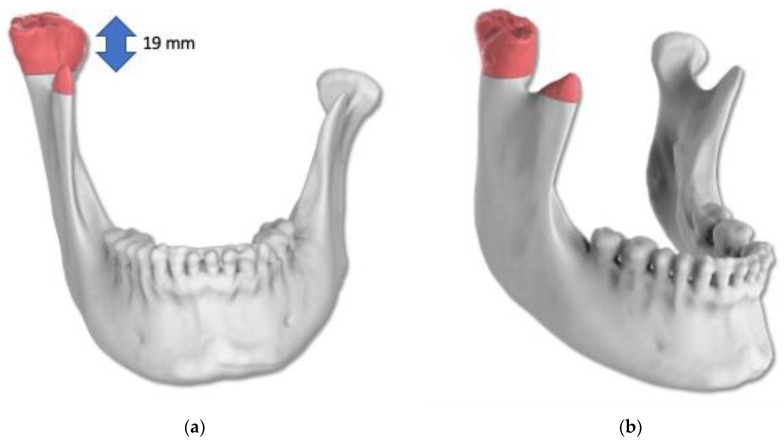
(**a**,**b**) Definition of the resection of the condyle and of the right coronoid. The length of the planned resection was 19 mm. The red parts show the definition of the resection of the condyle and of the right coronoid.

**Figure 4:**
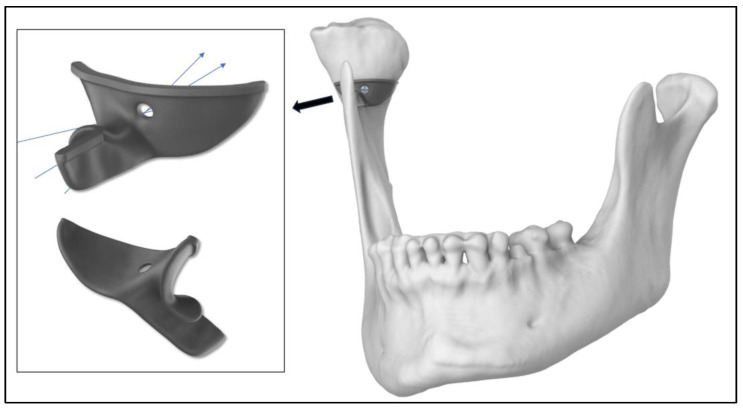
Titanium resection guide design was planned to be fixed with 1.5 mm screw. The template had a thickness of 0.9 mm with a 1.2 mm thick edge at the level of the resection. The unique design of the template with screw hole which allowed for a degree of angle of the easy screw placement. The blue arrows show the insertion and screwing angles of the fixation screws.

**Figure 5:**
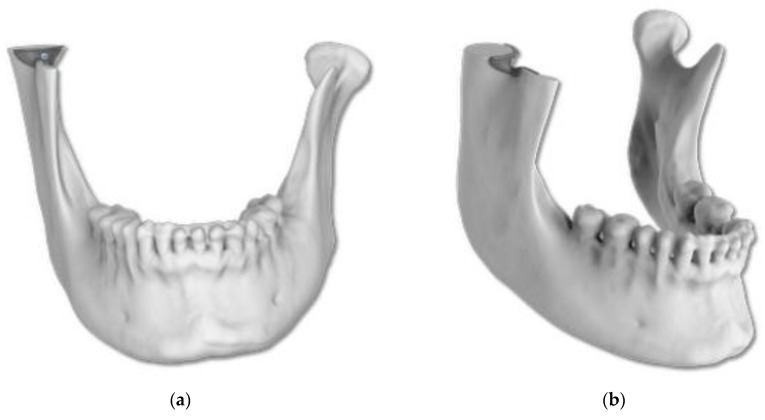
The planned resection of the condyle and the right coronoid from frontal (**a**,**b**) lateral views.

**Figure 6:**
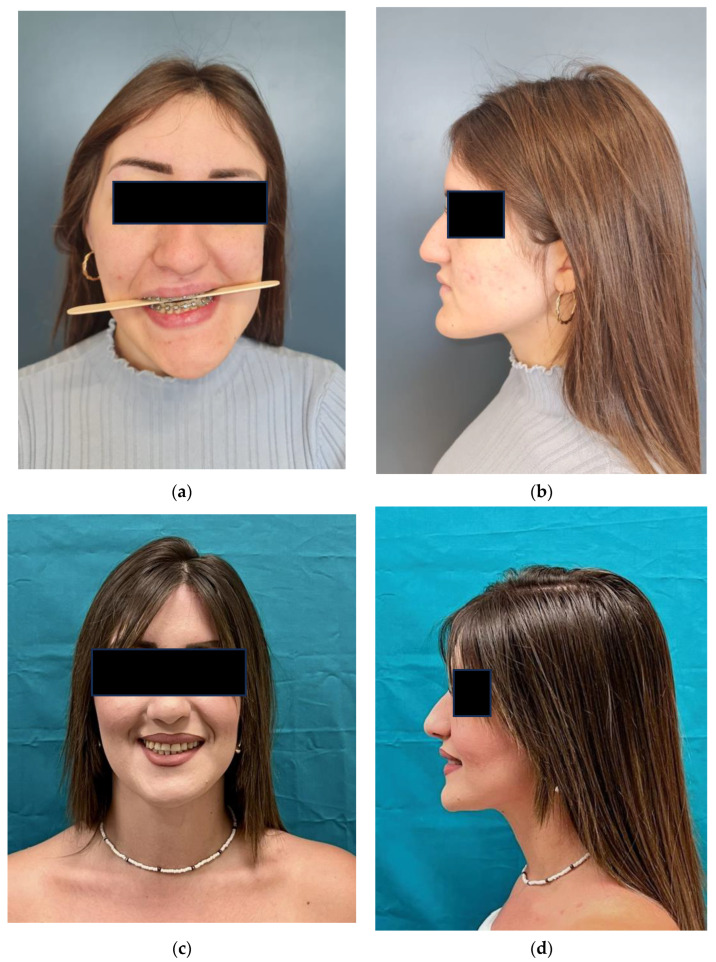
(**a**,**b**) Pre-operative and (**c**,**d**) post-operative photographs of the same patient showing improvements in terms of esthetics.

In order to test the protocol, the first two patients were operated in two sessions, condylectomy operations were done first, and orthognathic surgeries were done with 12–15 months intervals. The other five patients had condylectomy and orthognathic surgeries on the same day, otherwise the whole intra-oral condylectomy protocol was the same for all the patients. Orthognathic surgeries were done using protocols described by the same group of authors in a previous publication [24]. The planning of the condylectomy operation of the first patient can be seen in Figure 2, Figure 3, Figure 4, Figure 5 and Figure 6.

Condylectomy—Surgical Protocol:

Left/right condylectomy was performed under general anesthesia. After infiltration with local anesthetic added to a vasoconstrictor in the adherent mucosa of the lower vestibular fornix region, mucosal incision was done along the external oblique line and was extended to the left retromolar trigone. The surgery in brief was dissection, skeletonization of the left mandibular ramus, exposure of the coronoid process. Subsequent exposure of the anterior aspect of the left mandibular condyle with detachment of the joint capsule and pterygoid muscles; positioning of custom-made cutting guide using 1 screw, execution by guidance of osteotomy marks after positioning of the IMF (intermaxillary Fixation) screws at the level of the condylar head. Custom-made CAD/CAM cutting guide was positioned and condylectomy was performed along the osteotomy markings using Mectron Piezosurgery© (Mectron s.p.a., Carasco, Italy) ultrasonic instruments. The surgery continued with removal of the excess bone, including resection of the condyle and the coronoid process as planned (during coronoid process removal the reference point for removal was mandibular notch for all patients and no guide was utilized for this resection). After occlusal centric control and irrigation with saline solution, hemostasis was checked, the tissues were closed using 4.0 absorbable sutures (Vicryrl©, Ethicon (J&J Medtech Italia, Pomezia, Italy)). Orthopantomography and Massive-Facial CT (Cone Beam Tomography) was taken from the patient on the same day of the surgery for post-operative control.

Medications during the hospitalization:

Antibiotic therapy starting from a day before surgery for pre-operative prophylaxis and post-operative therapy (Amoxicillin/Clavulanic acid), analgesic therapy (Paracetamol, Ketorolac), anti-edema therapy (Dexamethasone), gastroprotective therapy (Pantoprazole), intravenous rehydration therapy with saline, anxiolytic therapy (Bromazepam).

The patients were hospitalized for 2 days starting from the day before surgery. Guide bands were replaced with occlusion maintained. Patients were in good general condition with optimum pain control. Surgical wounds were in order, there was no active bleeding, and no signs of infection. Edema, bruising and pain were compatible with date and mode of intervention. There was no deficit in sensitivity or local motility.

Recommendations for Home therapy:

Paracetamol 1 g tablet as needed (in case of fever or pain) after meals, maximum 1 tablet every 8 h. Antibiotic therapy for a week Amoxicillin/Clavulanic acid 875 + 125 mg, 1 sachet every 8 h. Maintenance of elastic intermaxillary block until the next checkup. Mouth opening exercises. Cold and soft diet for 3 days, followed by warm and soft diet for 15 days. Accurate oral hygiene immediately after meals with a toothbrush, toothpaste and mouthwash based on 0.2% diluted Chlorhexidine (maximum 10 days, then using a mouthwash without chlorhexidine).

Orthodontic evaluation was scheduled after a week by an orthodontist. Follow-up controls were scheduled as 7th day, after 1 month, 2 months and at 5th month.

Control follow-ups at 1st, 2nd, and 5th months

Routine check-up data that were obtained for all appointments were similar.

In brief: Regular course was same for all subjects. Edema progressively improving. No deficit in sensitivity or local motility. Good mandibular kinetics. Guide bands were removed and repositioned. Individual occlusion was maintained. Recommendations: maintenance of guide bands and mandibular gymnastic exercises as illustrated, and strict oral hygiene.

Cone Beam CT of the mandibular condyles was taken at 5 months after surgery. The patients had post-operative orthodontic therapy for adjustments in occlusion for 6 months. In the cases of the first two patients, orthognathic surgeries were planned at this stage.

Follow-up at 6 months following surgery

There were no complications during healing period and aesthetics were obtained with facial symmetry and good function. The patients represented no extra-oral scars on the face, due to the planned protocol via intra-oral approach which was achieved without encountering any problems.

The Supplementary Figures S1–S9 show the whole protocol including virtual planning for operations and intra-operative/post-operative photos of the third patient that had surgeries in one session.

The Supplementary Figures S10–S15 show virtual planning for operations and intra-operative/post-operative photos of another patient that had surgeries in two sessions.

The supplementary Figures S16–S25 show another case with the steps of all protocol with intra-operative/post-operative photos and CBCTs.

## 3. Results

In this study, no statistical analyses were done due to small number of the subjects, and the descriptive results regarding patients are presented in Table 1. The accuracy of the planned surgeries was not measured in this pilot study because it was not the scope of this research.

## 4. Discussion

The early treatment of UCH in early adolescents using proportional condylectomy does not commonly require a secondary orthognathic surgery [21]; however, in adult patients, traditional approach for management of UCH is the combination of orthodontics and condylectomy surgeries that are reported by several successful reports [3,5,10]. Early diagnosis is very important for successful management of UCH and patient records with serial photographs to follow the prognosis can be very critical at this point [8,25,26].

Currently, there are a very limited number of reports in the literature on management of unilateral condylar hyperplasia patients using intra-oral minimally invasive virtual methods [3,5,10]. Hernández-Alfaro et al. in 2016 introduced a novel minimally invasive technique for condylectomy performed through an intra-oral approach based on precise three-dimensional virtual planning [10]. Their study included seven consecutive patients treated using 3D-printed cutting guides to define operative references, which provided excellent access for total or partial condylectomy through a limited intra-oral incision. They additionally utilized piezoelectric instruments for increased safety. According to their experience, surgical time reduced to an average of 16.9 min, and post-operative morbidity was found to be minimal [10]. In 2020, the same team of authors published another report on the same technique in more detail than they had previously described [5]. The technique consisted of two steps: virtual surgical planning and intra-oral condylectomy. During virtual surgical planning, the mandibular ramus was measured bilaterally, the height of the proportional condylectomy was planned virtually, and a cutting guide was 3D-printed. In the surgery, the mandibular condyle was approached intra-orally, the 3D-printed cutting guide was positioned in the sigmoid notch, and the proportional condylectomy was performed with success [5].

In a more recent similar study that was published in 2022, Yuan et al. [3] introduced an innovative method for mandibular condylectomy using a custom-made three-dimensional 3D-printed template. In their case report on a single patient, condylectomy combined with orthognathic surgery was used for the treatment of facial asymmetry secondary to unilateral condylar hyperplasia. In brief, the 3D-printed guide was placed using intra-oral approach, and the condylectomy was performed. The osteotomy line was accurately transferred from the virtual surgical plan to the real surgery, and as a result they reported successful outcomes [3]. As an advantage, the conventional pre-auricular extraoral incision was avoided, which has a potential risk of facial nerve injury and inevitable facial scarring [3]. In their study, Yuan et al. performed condylectomy combined with orthognathic surgery in a single session [3]. However, the protocol described in this paper involved mandibular condylectomy and orthognathic surgery in two sessions (for the first two patients) as condylectomy operation first, followed by orthognathic surgery after a break of one year for two patients. The main reason for a period of waiting of one year between two surgeries was to observe and evaluate the adaptation condylar remodeling as post-proportional condylectomy. The second orthognathic surgery provided further aesthetics, which were supported by orthodontic therapy for six months. The following patients had both surgeries, which included condylar operation followed by bimaxillary orthognathic surgery, in the same session.

The surgical protocol that is described in this study is very similar to the methods described by these three reports mentioned above as they have investigated innovative methods for mandibular condylectomy using a custom-made 3D-printed template [3,5,10]. However, they used condylar cutting guides made of different materials such as acrylic, while in this study titanium was used. Additionally, the surgical time we experienced was much longer than that which was reported by Hernández-Alfaro; it was even much shorter when compared to other conventional condylectomy surgical operations via extra-oral approach [8]. As the authors of this work experienced approximately 30 min for the total timing of the surgery (double the time by more than 15–16 min that was mentioned by Hernández-Alfaro) [10].

The intra-oral protocol presented in this paper presents some technical difficulties and increased costs. Firstly, the restricted access to the surgical field is the main challenge. This limited access and visibility of such a complex anatomical structure increases the difficulty of the operation. So, it would be advisable that such surgeries are performed by experienced operators. Facial and auriculotemporal nerve injury is less when compared to extra-oral approach, but in both protocols, there is always a possibility of vascular or nerve injury. It would be recommended to utilize special tools such as piezoelectric surgery instruments to prevent any soft tissue damage and to obtain results with increased accuracy. At this point, the guide used in this work seemed to facilitate the precise excision of the excess mandibular parts as was planned before surgery. The associated costs when compared to traditional methods were higher because the patient-specific guides had to be produced using expensive virtual methods. However, it was acceptable and preferred by the patients because of the advantages such as better aesthetics and minor risks. Another point that the originality of this paper relies on is the use of titanium cutting guides. Both acrylic and titanium cutting guides are biocompatible and widely used in maxillofacial operations. However, titanium guides represent advantages such as improved rigidity with resistance to deformation and high precision as they are less prone to flexing during cutting or drilling [27]. Additionally, synthetic polymer guides are generally thicker and need more tissue reflection, while titanium guides have a thinner profile which makes the placement easier with smaller incisions (which is especially advantageous in our cases that have restricted access and visibility to the surgical field).

In this present study, two patients were operated on in two separate sessions, while the other five patients were operated on as condylectomy and orthognathic surgery on the same day. In patients with unilateral mandibular condylar hyperplasia, whether to perform condylectomy and orthognathic surgical procedures on the same day or in two stages remains controversial [20]. One-stage simultaneous surgery is usually preferred in active SPECT + adult patients. Instead, two-stage surgery is mostly considered in cases of skeletally immature patients with mild initial deformity to control and observe the growth [20,25,26,28]. However, this approach presents some disadvantages such as between the two surgeries the patient remains asymmetric and total time of treatment is longer. Furthermore, two different sessions of general anesthesia can represent some risks. On the other hand, in cases of simultaneous surgery, there are concerns such as long-term skeletal stability, absence of a remodeling interval, and the lack of standardized selection criteria [29]. Additional risk to mention is post-operative relapse, so each case should be evaluated by the patient-specific factors that might affect the outcomes of the surgeries. In this study, all the patients included were adults (except patient 2, who was operated on in two sessions), and the deformities were moderate to severe. The decision of two stages or one simply depended on the decision of the surgeon to test the intra-oral protocol represented.

Each unilateral mandibular condylar hyperplasia case should be evaluated for the patient-specific factors that will affect the outcomes of the surgeries. There is still a limited number of papers in the literature on this topic and further comparative clinic research should be done to understand the advantages and disadvantages of each in order to choose the best option in different scenarios. At this point, limitations of this study include small sample size with no comparison group (no comparison groups for one/two-stage surgeries and extra-oral and intra-oral approach) and short follow-up period. However, there is a great lack in the literature on the intra-oral management of unilateral mandibular condylar hyperplasia and, when compared to other reports, this preliminary report has value as it is on seven cases as a part of ongoing research. Further limitations of this study include no evaluation of the surgeries for accuracy of the planning with the outcomes obtained.

## 5. Conclusions

As a conclusion, according to the results of this study and previous publications, intra-oral condylectomy operations with 3D-printed titanium cutting guides can be considered as a less invasive alternative approach with promising successful outcomes such as improved esthetics and no risks of facial nerve injuries for the treatment of mandibular condylar hyperplasia patients. However, further studies should be conducted with a larger number of subjects in combination with orthognathic surgeries (simultaneous or in two sessions) to confirm these results. Specially, long-term results of this kind of surgery are critical to assessing the stability of the facial symmetry obtained.

## Figures and Tables

**Table 1:** Details about surgeries, patient information and demographics.

	Patient 1	Patient 2	Patient 3	Patient 4	Patient 5	Patient 6	Patient 7
Gender	Female	Female	Female	Male	Female	Male	Female
UCH	Right	Left	Left	Right	Right	Right	Left
Age *	25	17	26	27	27	30	33
UCH Height Difference (mm)	19	6	7	10.1	13.6	9.2	4.1
SPECT R (%)	57	42	44	55	69	60	41
SPECT L (%)	35	56	55	45	54	47	53
SPECT (%) differences	22	14	11	10	15	13	12
Surgeries and timing	12-month interval	14-month interval	One session	One session	One session	One session	One session
Genioplasty	Yes	Yes	No	No	Yes	No	No
Lipofilling	No	Yes	No	No	No	No	No
Total Follow-up (months)	24	23	17	7	4	2	1

UCH: Unilateral mandibular condyle with condylar hyperplasia, SPECT: Single photon emission computed tomography, * Age at first visit.

## Data Availability

The raw data supporting the conclusions of this article will be made available by the authors on request.

## Supplementary Materials

The following supporting information can be downloaded at: https://www.mdpi.com/article/10.3390/jcm15072671/s1.

## Abbreviations

The following abbreviations are used in this manuscript:

UCH	Unilateral condylar hyperplasia
SPECT	Single photon emission computed tomography
MCH	Mandibular condylar hyperplasia
IMF	Intermaxillary Fixation
CAD/CAM	Computer-aided design and computer-aided manufacturing
STL	Standard Tessellation Language
