# Supplementary material for: Minimally Invasive Protocol for the Management of Unilateral Condylar Hyperplasia: Case Series on Seven Patients
Source: J Clin Med. 2026 Apr 1;15(7):2671. doi: 10.3390/jcm15072671 (PMC13074066; doi:10.3390/jcm15072671)
Supplement: Supplementary file 1 [file jcm-15-02671-s001.zip › jcm-4208866-supplementary.pdf]

The Supplementary Figures S1–S9: The Supplementary Figures S1-9 show the whole protocol including virtual planning for operations and intra-operative / post-operative photos of the third patient that had surgeries in one session.

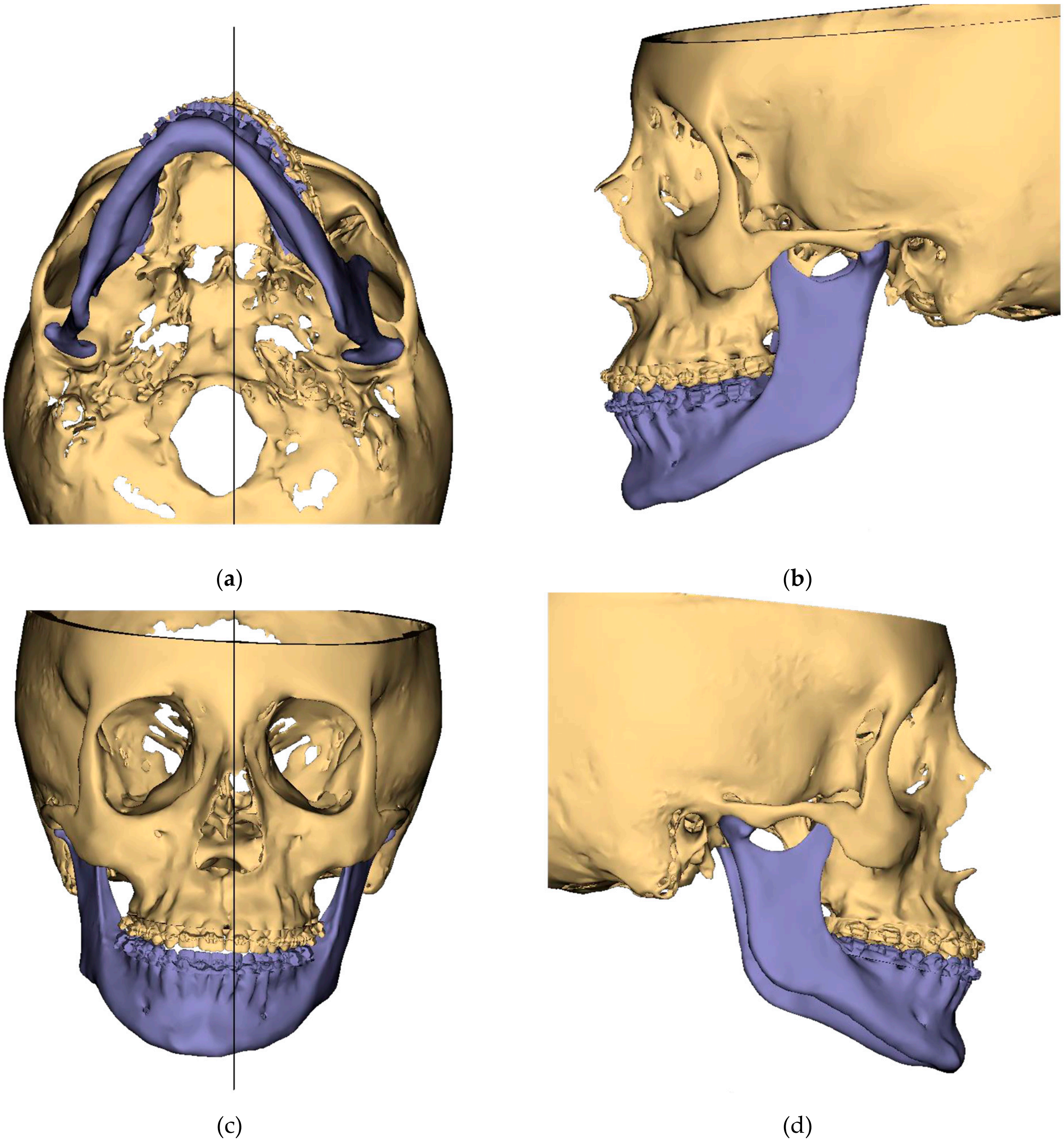

Figure S1. Surgical Plan: Pre-operative Position (**a**) Pre-operative situation of the patient showing asymmetry; (**b,c,d**) Pre-operative situation from lateral left (b), frontal (c) and lateral right sides.

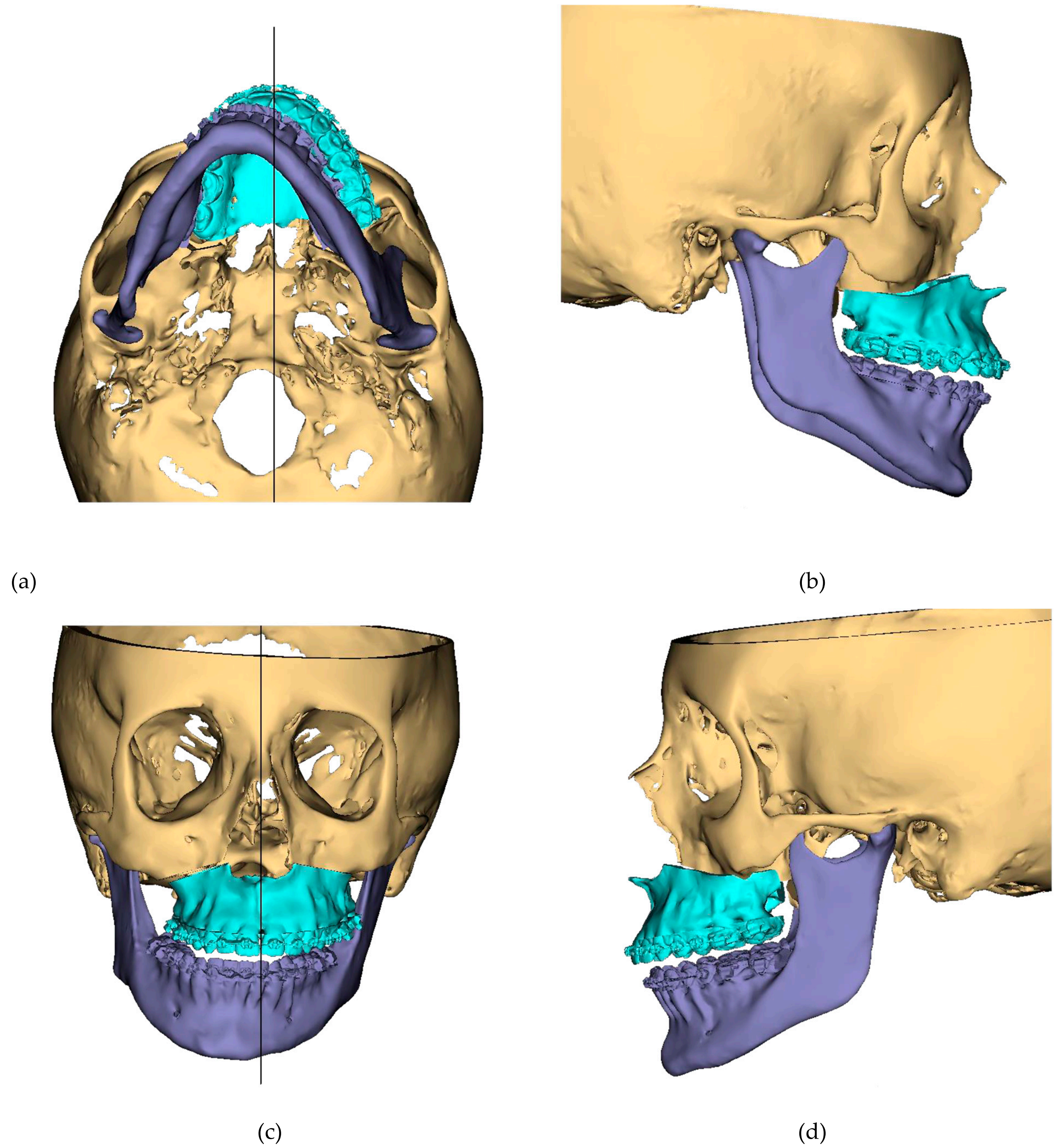

Figure S2. Surgical Plan: Intermediate Position (**a**) Planning for Maxillary movement first; (**b**,**c**,**d**) Pre-operative planning for maxillary movement from lateral left (b), frontal (c) and lateral right sides.

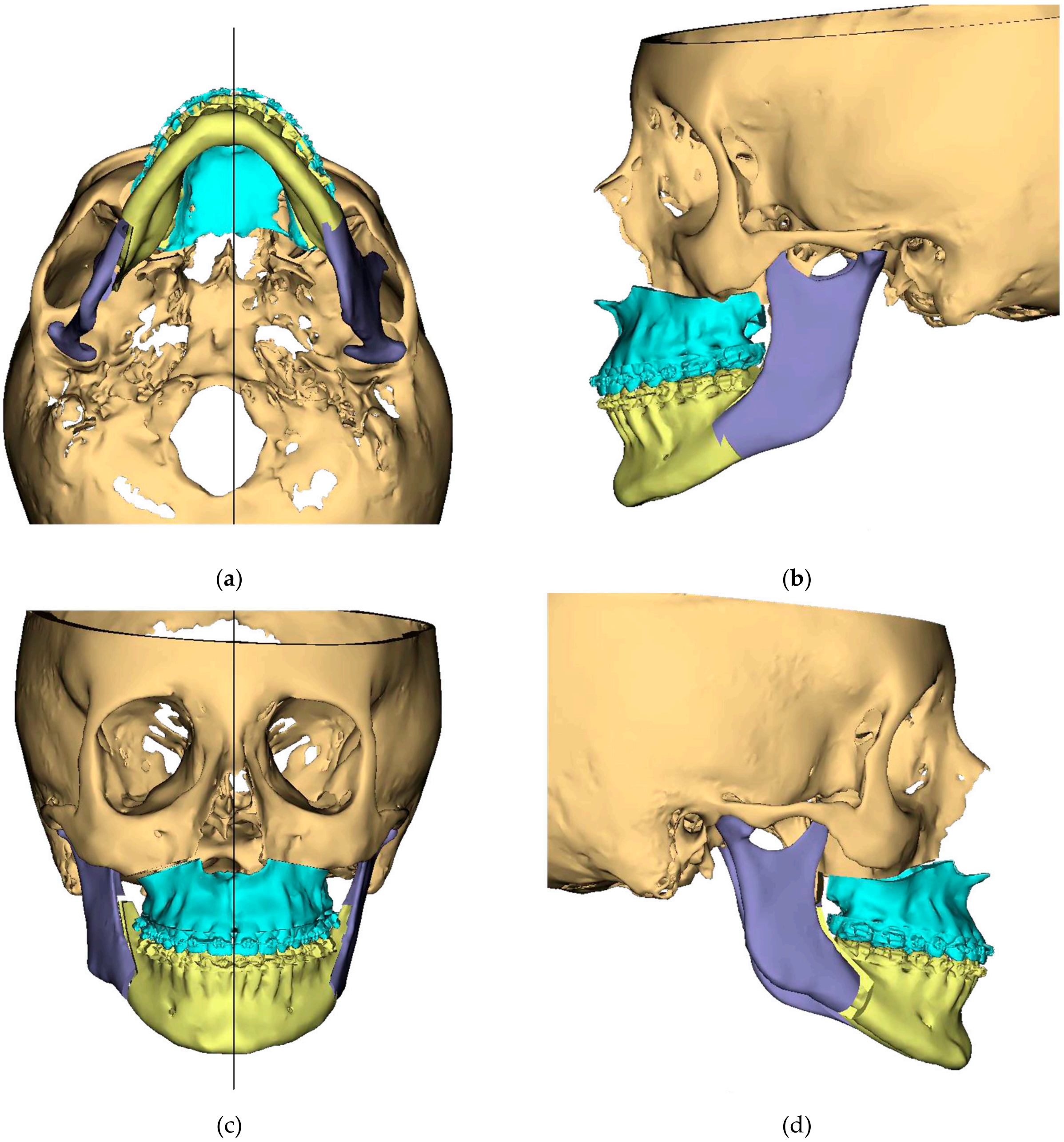

Figure S3. Surgical Plan: Final Position (**a**) Planning for mandibular position; (**b,c,d**) Pre-operative planning for sagittal-splint osteotomies of the mandibular bone maxillary movement from lateral left (b), frontal (c) and lateral right sides.

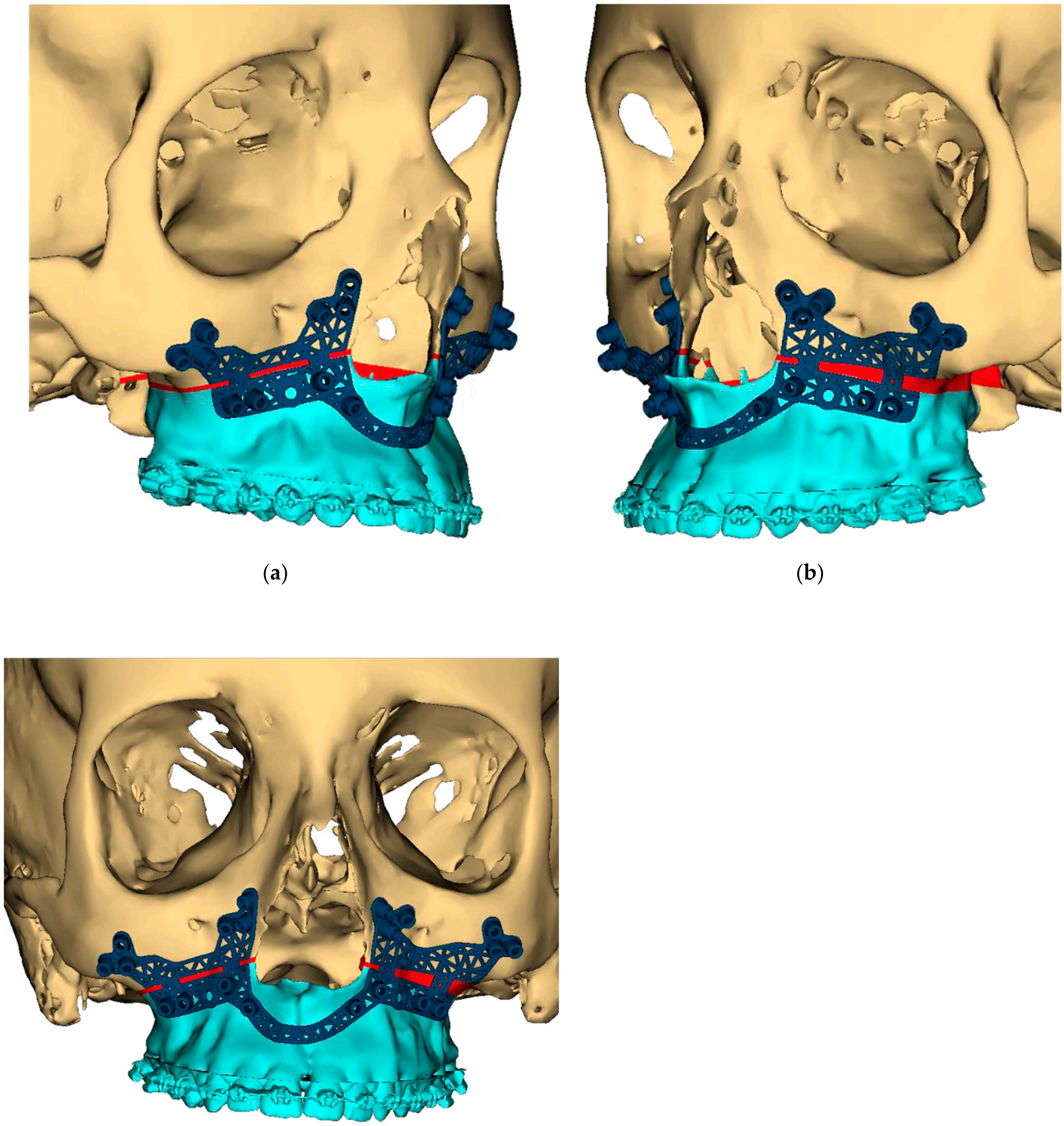

(**a**) (**b**)

(c)

Figure S4. Cutting Guide Design: CAD/CAM Titanium 3D Printed Guide for Midface**.** (**a**) Planning for maxillary osteotomies using cutting guide from frontal view. (**b,c**) pre-operative planning for cutting guide from lateral left (b) and and lateral right (c) sides.

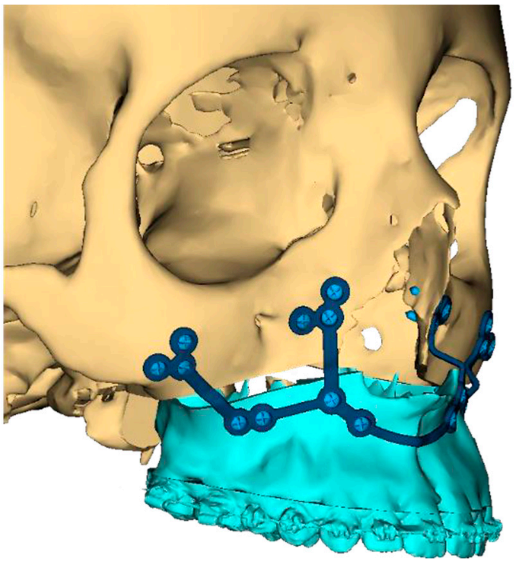

(**a**)

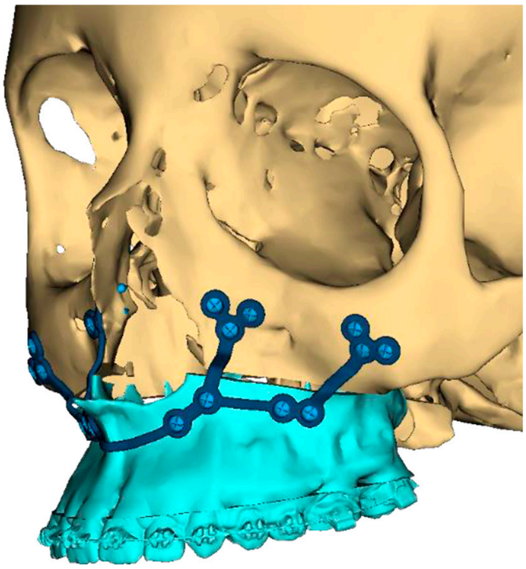

(**b**)

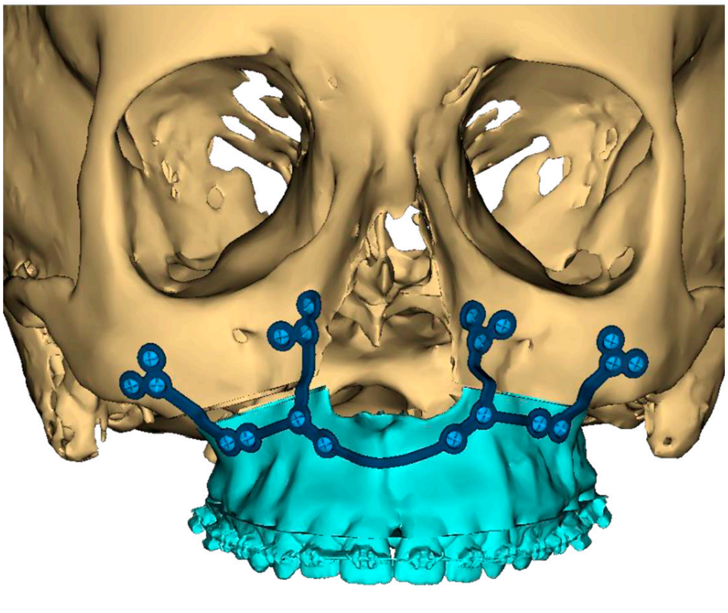

(c)

Figure S5. Titanium plate design for fixing maxillary bone for its final postion: Materialise Titanium 3D Printed Plate for Midface (1 mm thickness) Screw holes were for use with MatrixORTHOGNATHIC Ø1.85 mm screws. All screws were pre-drilled using surgical guides. Maxillary positioning was planned for using fixing plate with 20 screws. (**a**,**b**,**c**) pre-operative planning for maxillary position with 2.2mm and 1.8 mm gaps on left side and 3.3mm and 0.6 mm overlapping on right side. Views from lateral left (a), lateral right (b) and frontal.

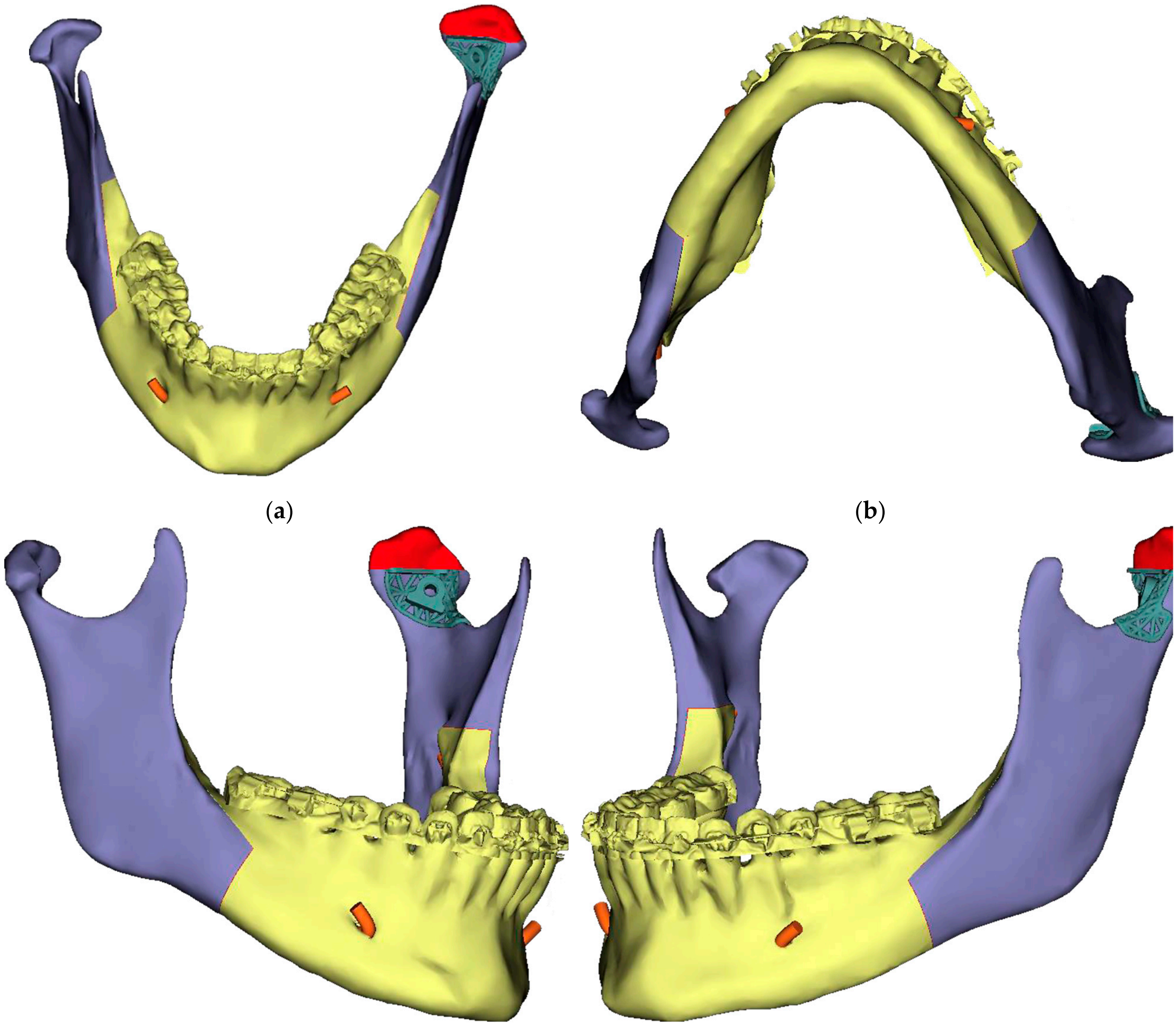

(**a**) (**b**)

(c) (d)

Figure S6. Titanium 3D printed guide for condylar resection surgery on left side. (**a**,**b**,**c**,**d**) Condylar cutting guide from occlusal and lateral views. Note: Blue: Fixation hole (temporary fixation of guide- indicated in blue) for use with MatrixORTHOGNATHIC Ø1.85 mm screws → Red color shows planned resected mandible and mental nerve position.

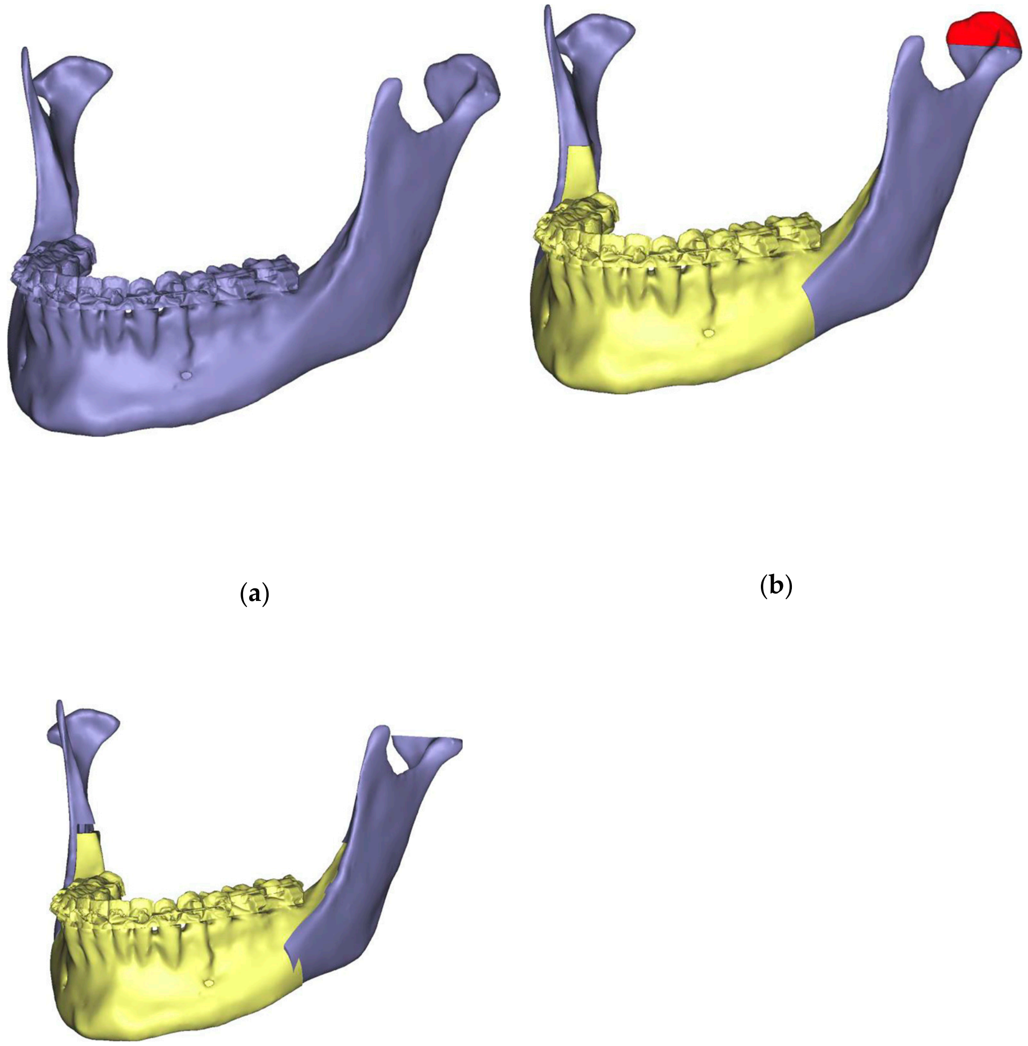

(**a**) (**b**)

(c)

Figure S7. Pre-operative situation for condylar osteotomy planning (**a**) pre-operative, (**b**) post-operative planned resection of the mandibular condyle (Red color), (**c**) resected mandible as planned.

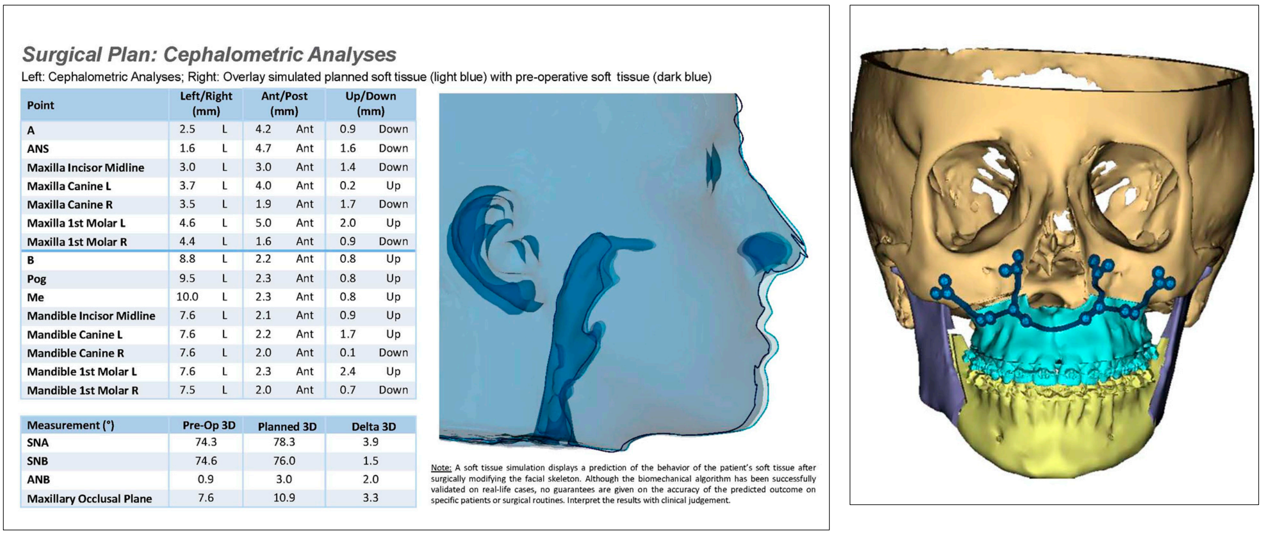

Surgical Plan: Cephalometric Analyses

Left: Cephalometric Analyses; Right: Overlay simulated planned soft tissue (light blue) with pre-operative soft tissue (dark blue)

Point	Left/Right (mm)		Ant/Post (mm)		Up/Down (mm)	
A	2.5	L	4.2	Ant	0.9	Down
ANS	1.6	L	4.7	Ant	1.6	Down
Maxilla Incisor Midline	3.0	L	3.0	Ant	1.4	Down
Maxilla Canine L	3.7	L	4.0	Ant	0.2	Up
Maxilla Canine R	3.5	L	1.9	Ant	1.7	Down
Maxilla 1st Molar L	4.6	L	5.0	Ant	2.0	Up
Maxilla 1st Molar R	4.4	L	1.6	Ant	0.9	Down
B	8.8	L	2.2	Ant	0.8	Up
Pog	9.5	L	2.3	Ant	0.8	Up
Me	10.0	L	2.3	Ant	0.8	Up
Mandible Incisor Midline	7.6	L	2.1	Ant	0.9	Up
Mandible Canine L	7.6	L	2.2	Ant	1.7	Up
Mandible Canine R	7.6	L	2.0	Ant	0.1	Down
Mandible 1st Molar L	7.6	L	2.3	Ant	2.4	Up
Mandible 1st Molar R	7.5	L	2.0	Ant	0.7	Down

Measurement (°)	Pre-Op 3D	Planned 3D	Delta 3D
SNA	74.3	78.3	3.9
SNB	74.6	76.0	1.5
ANB	0.9	3.0	2.0
Maxillary Occlusal Plane	7.6	10.9	3.3

Note: A soft tissue simulation displays a prediction of the behavior of the patient's soft tissue after surgically modifying the facial skeleton. Although the biomechanical algorithm has been successfully validated on real-life cases, no guarantees are given on the accuracy of the predicted outcome on specific patients or surgical routines. Interpret the results with clinical judgement.

(**a**) (**b**)

Figure S8. (**a**) Cephalometric analyses (**b**) Planned final situation. Cephalometric analyses reference measurements are listed as: A (deepest point of concavity on the anterior surface of the maxilla), ANS (Anterior nasal spine), maxilla incisor midline, maxilla canine left&right, maxilla 1st molar left&right, B, Pogonion, Menton, mandibular incisor midline, mandibular canine left&right, mandibular 1st molar left&right. SNA angle (angle of Sella, Nasion and A), SNB angle (angle of Sella, Nasion and B), ANB angle (angle of a, Nasion and B points).

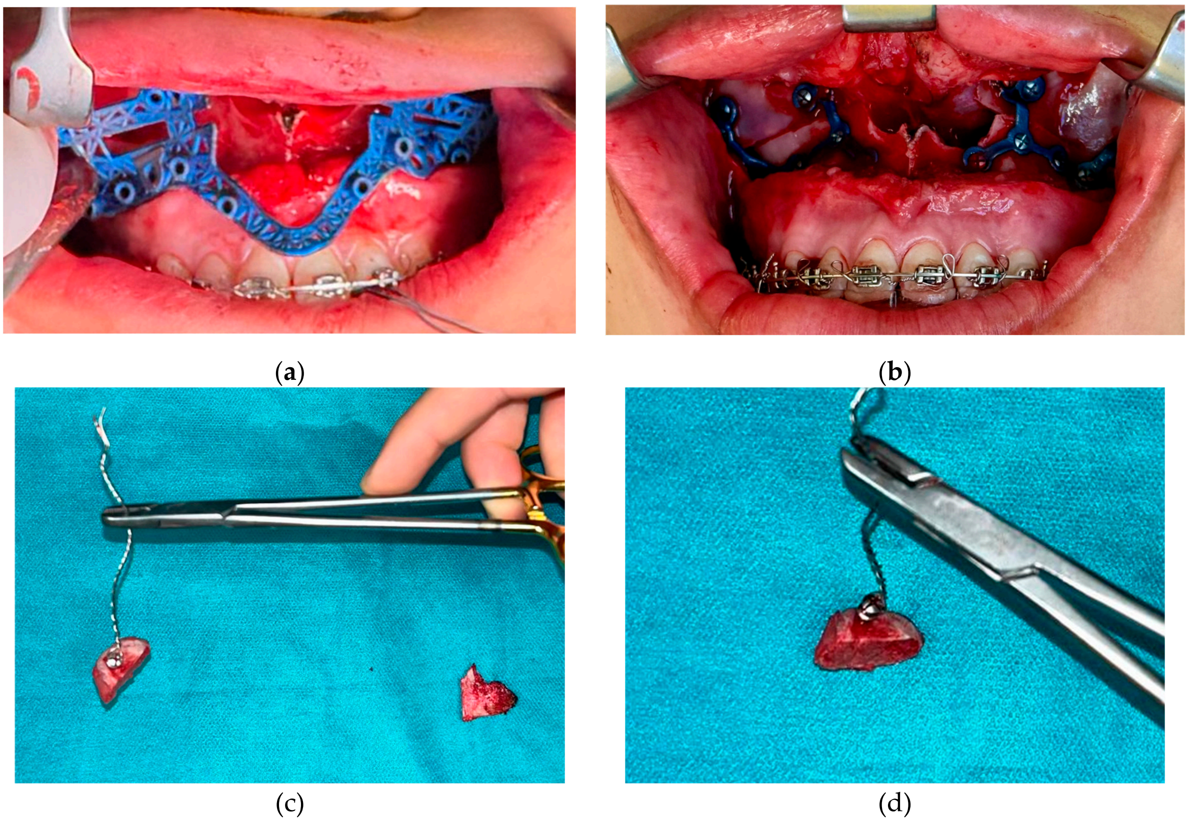

Figure S9. Intra-operative photos showing (a) maxillary cutting guide, (b) maxillary fixation plate (c, d) Mandibular resected condyle.

Supplementary Figures S10–S15: The virtual planning for operations and intra-operative / post-operative photos of the second patient that had surgeries in two sessions

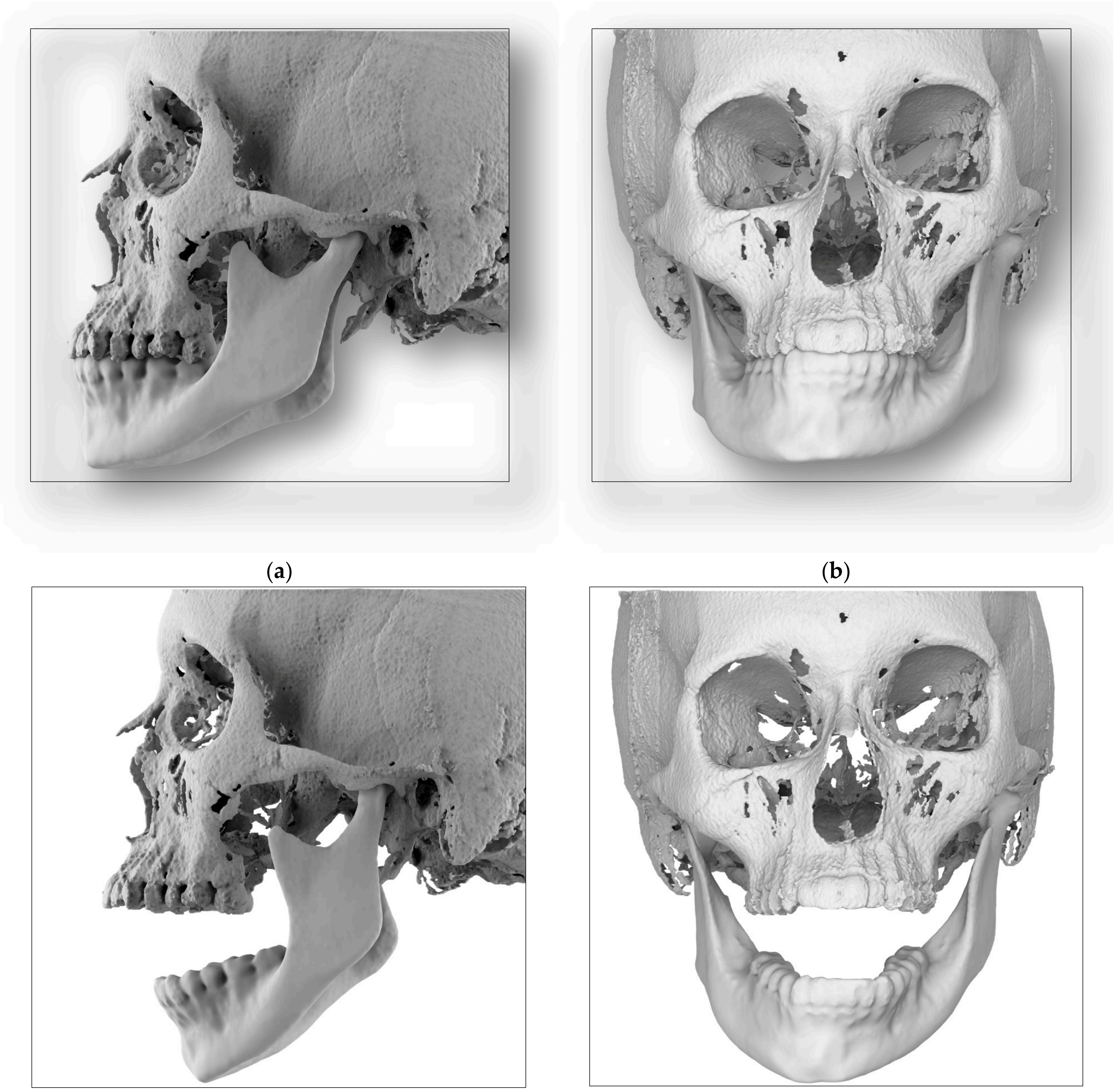

(**a**) (**b**) (c) (d)

Figure S10: Pre-operative situation with asymmetry. **(a)** Pre-operative profile; **(b)** frontal situation with mouth closed; **(c)** pre-operative situation at mouth opening from profile view; **(d)** and frontal view showing deviation.

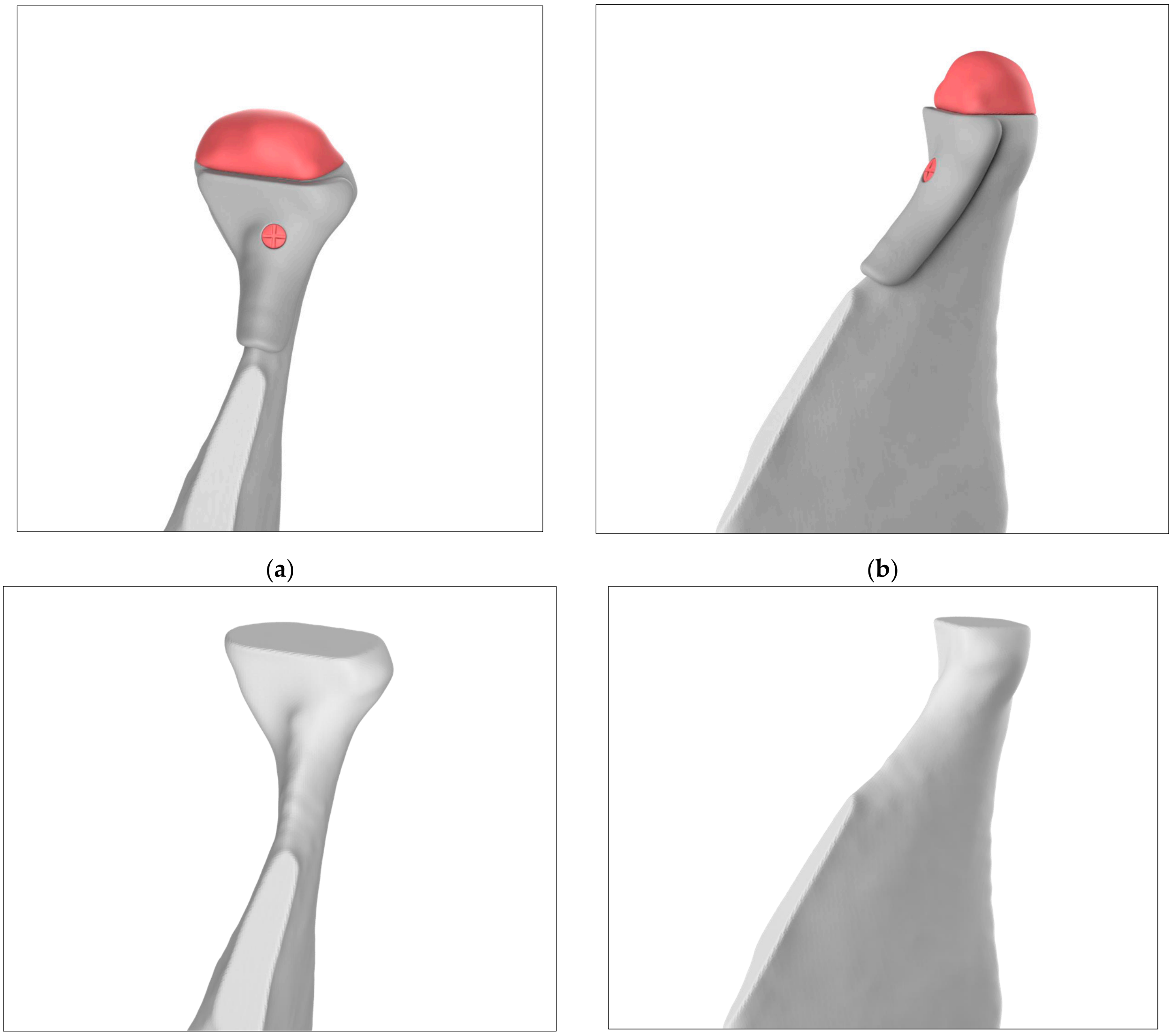

Figure S11: Pre-operative planning for removal of the excess condyle using CAD\CAM methods. **(a-b)** Titanium resection guide design fixed with a 1.5 mm screw. The template to be placed 6mm from the apex of the condyle. Figures a and b highlighting the excess bone volume and removal of the excess bone; **(c-d)** Representative figure showing planning of removal of the excess condyle.

Kit reference	N/A	
Item reference	ID	Preview
CMF.Ti074D	I1	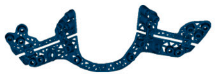
CMF.Ti054D	G1	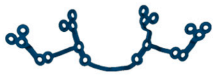
CMF.Ti070D	I2	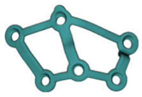
CMF.Ti053D	G2	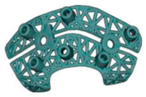

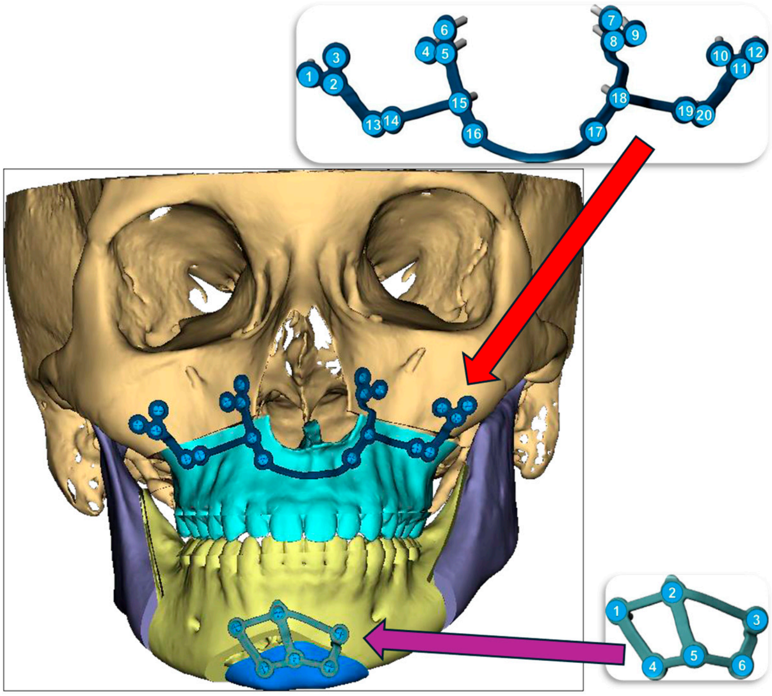

Figure S12: Summary of Delivered Personalized Medical Devices (Recommended screws were 6.0 mm length\ 1.85mm diameter\ Self-Tapping\ 04.511.206.01C\ MatrixORTHOGNATHIC). Fixation with 26 screws (20 for maxilla showed with red arrow and 6 for mandible indicated with violet colored arrow) in total were planned. (Screws Specifications for use with Titanium 3D Printed Plate for Midface: **Hole ID \Length*(mm)\ Diameter (mm)**\ **Style**\ **Articlenumber**\ **Platingsystem**\ **Position**: **1-12:** 6mm length\ diam 1.85mm\ Self-Tapping\ 04.511.206.01C\ MatrixORTHOGNATHIC\ Skull Remaining. **13-20:** 6.0mm length\ 1.85 mm diameter \Self-Tapping \04.511.206.01C\ MatrixORTHOGNATHIC \Maxilla). *(*Plate Design: MaterialiseTitanium 3D Printed Plate for Mandible (1 mm): Screw hole for use with MatrixORTHOGNATHIC Ø1.85 mm screws. Hole ID \Length*(mm)\ Diameter (mm)\ Style\ Articlenumber\ Platingsystem\ Position **1-3:** 6.0mm L\ 1.85mm diam\ Self-Tapping \04.511.206.01C\ MatrixORTHOGNATHIC \Mandible Body. **4-6: 6.0**\ 1.85\ Self-Tapping\ 04.511.206.01C \MatrixORTHOGNATHIC \Chin. Note: All screw pre-drilling guided using surgical guides.

Point	Left/Right (mm)		Ant/Post (mm)		Up/Down (mm)	
**A**	3.0	L	3.0	Ant	0.6	Up
**ANS**	3.3	L	2.9	Ant	0.6	Up
**Maxilla Incisor Midline**	2.5	L	3.0	Ant	0.7	Up
**Maxilla Canine L**	2.1	L	2.3	Ant	0.0	N/A
**Maxilla Canine R**	2.0	L	3.9	Ant	1.5	Up
**Maxilla 1st Molar L**	1.3	L	2.0	Ant	0.4	Down
**Maxilla 1st Molar R**	1.2	L	4.2	Ant	1.9	Up
**B**	0.2	L	7.0	Ant	0.6	Up
**Pog**	4.8	L	8.9	Ant	1.6	Down
**Me**	4.0	L	7.9	Ant	1.0	Down
**Mandible Incisor Midline**	1.0	L	7.1	Ant	0.3	Up
**Mandible Canine L**	0.9	L	6.6	Ant	0.1	Down
**Mandible Canine R**	0.8	L	7.5	Ant	1.0	Up
**Mandible 1st Molar L**	0.2	L	6.3	Ant	0.2	Down
**Mandible 1st Molar R**	0.1	L	7.8	Ant	1.6	Up

Measurement (°)	Pre-Op 3D	Planned 3D	Delta 3D
**SNA**	85.5	88.2	2.8
**SNB**	80.5	84.7	4.2
**ANB**	5.7	6.4	0.7
**Maxillary Occlusal Plane**	6.5	7.4	0.9

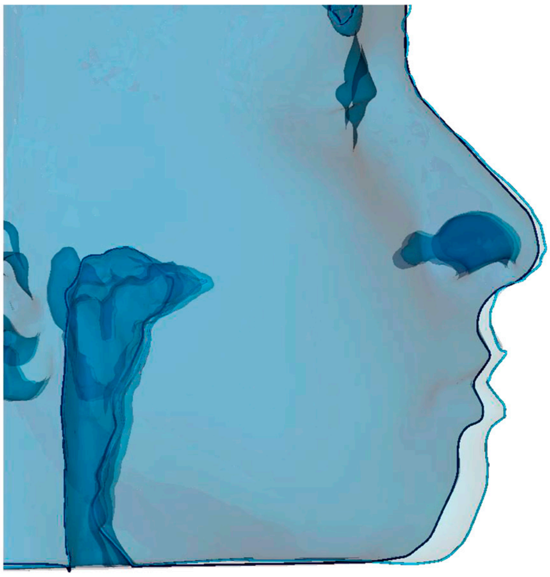

(**a**)

Figure S13: Surgical Plan with Cephalometric Analyses [Left: Cephalometric Analyses; Right: Overlay simulated planned soft tissue (light blue) with pre-operative soft tissue (dark blue)].

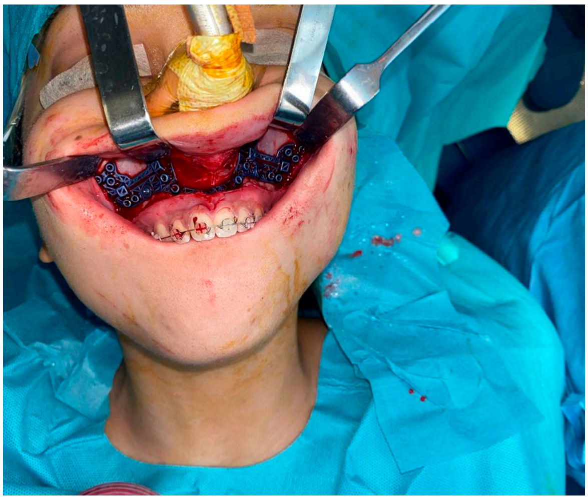

(**a**)

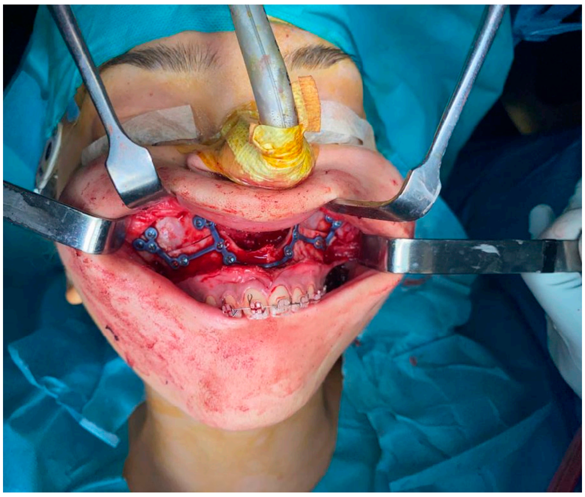

(**b**)

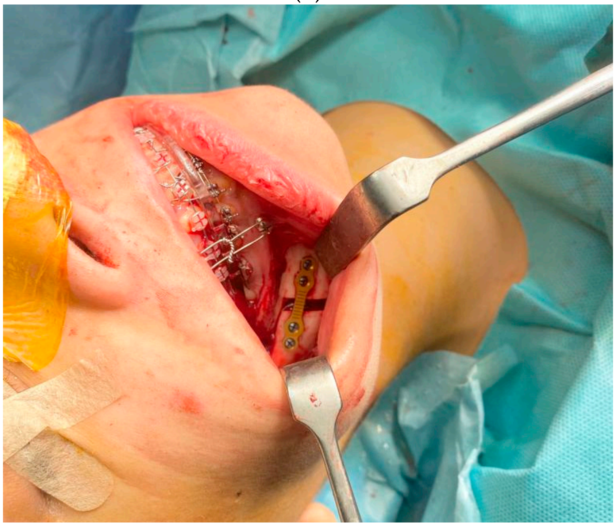

(c)

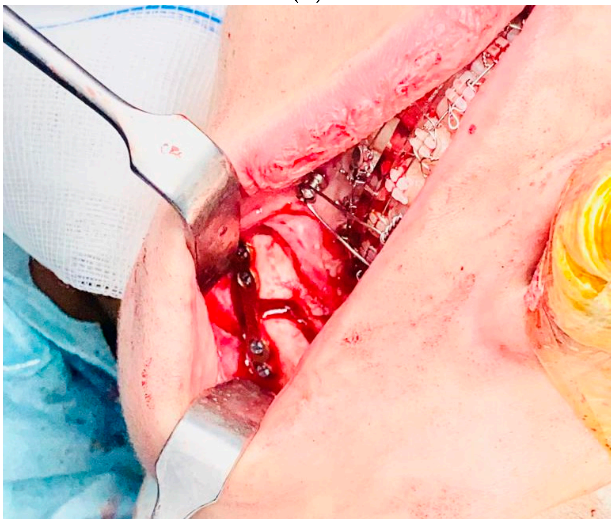

(d)

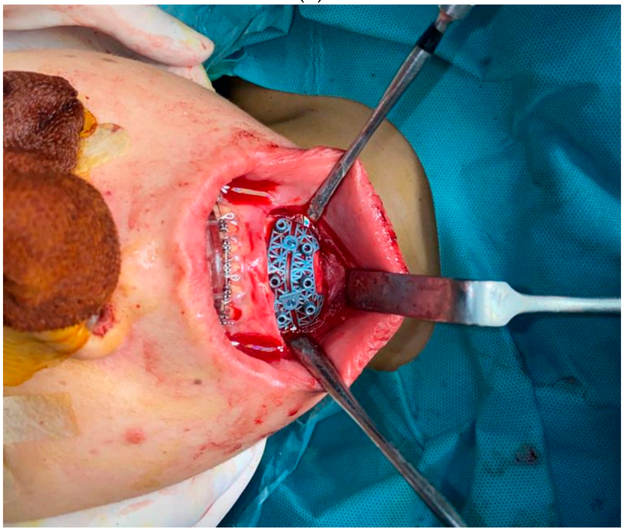

(e)

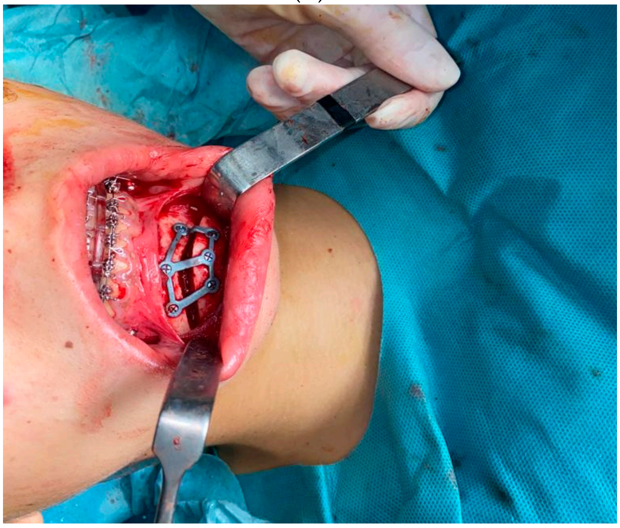

(f)

Figure S14: (a) Intra-operational view of the patient showing Materialise Titanium 3D Printed Guide for Midface cutting guide; **(b)** Intra-operational view of the patient showing MaterialiseTitanium 3D Printed Plate for Midface (1 mm); **(c)** Fixation plate with 4 screws for repositioning of the right side of the mandible; **(d)** Plate with 4 screws for repositioning of the left side of the mandible; **(e)** Intra-operational view of the patient showing Materialise Titanium 3D Printed Guide for genioplasty cutting guide; **(f)** Intra-operational view of the patient showing MaterialiseTitanium 3D Printed Plate placed on chin of the patient.

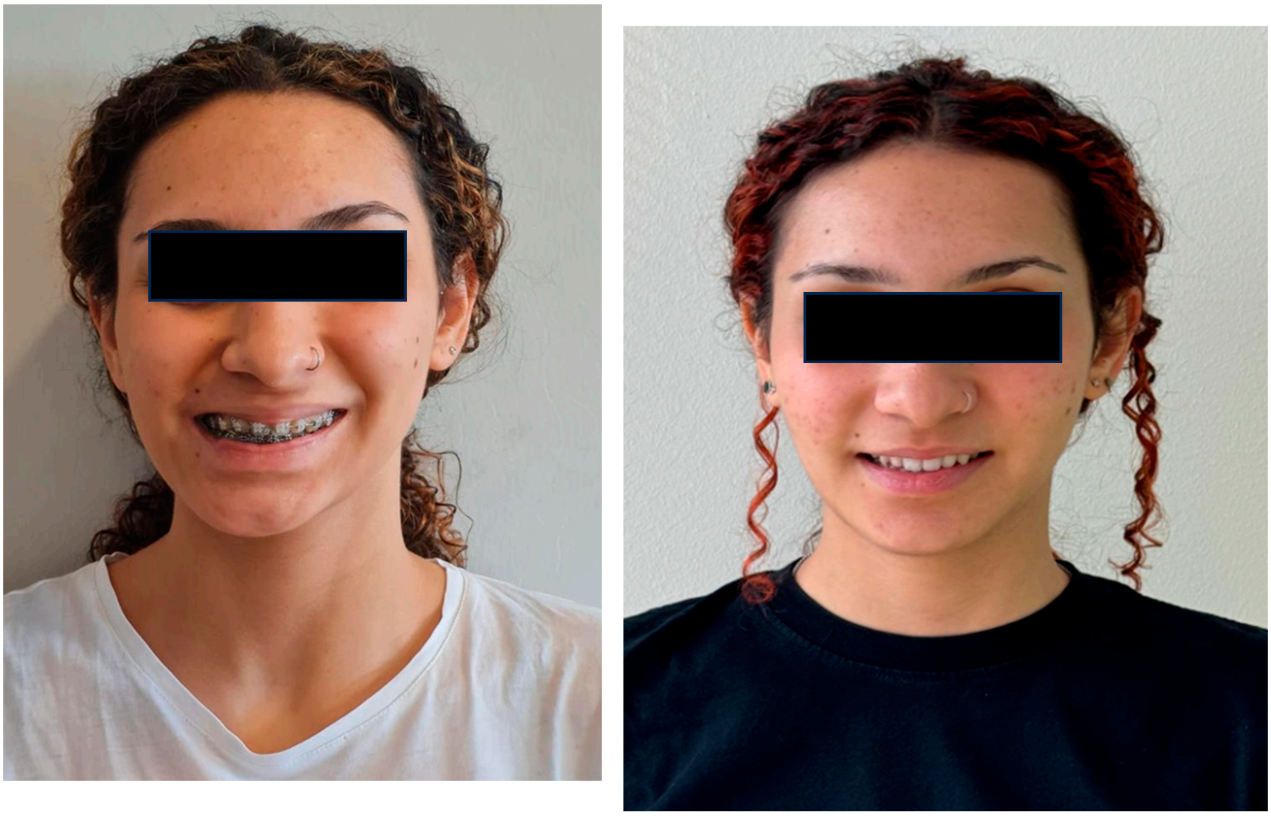

(**a**) (**b**)

Figure S15: (a) Pre-operative and **(b)** Post-operative photographs of the same patient.

The Supplementary Figures S16–S25: These Supplementary Figures show the whole protocol including virtual planning for operations and intra-operative / post-operative photos of another patient that had surgeries in one session.

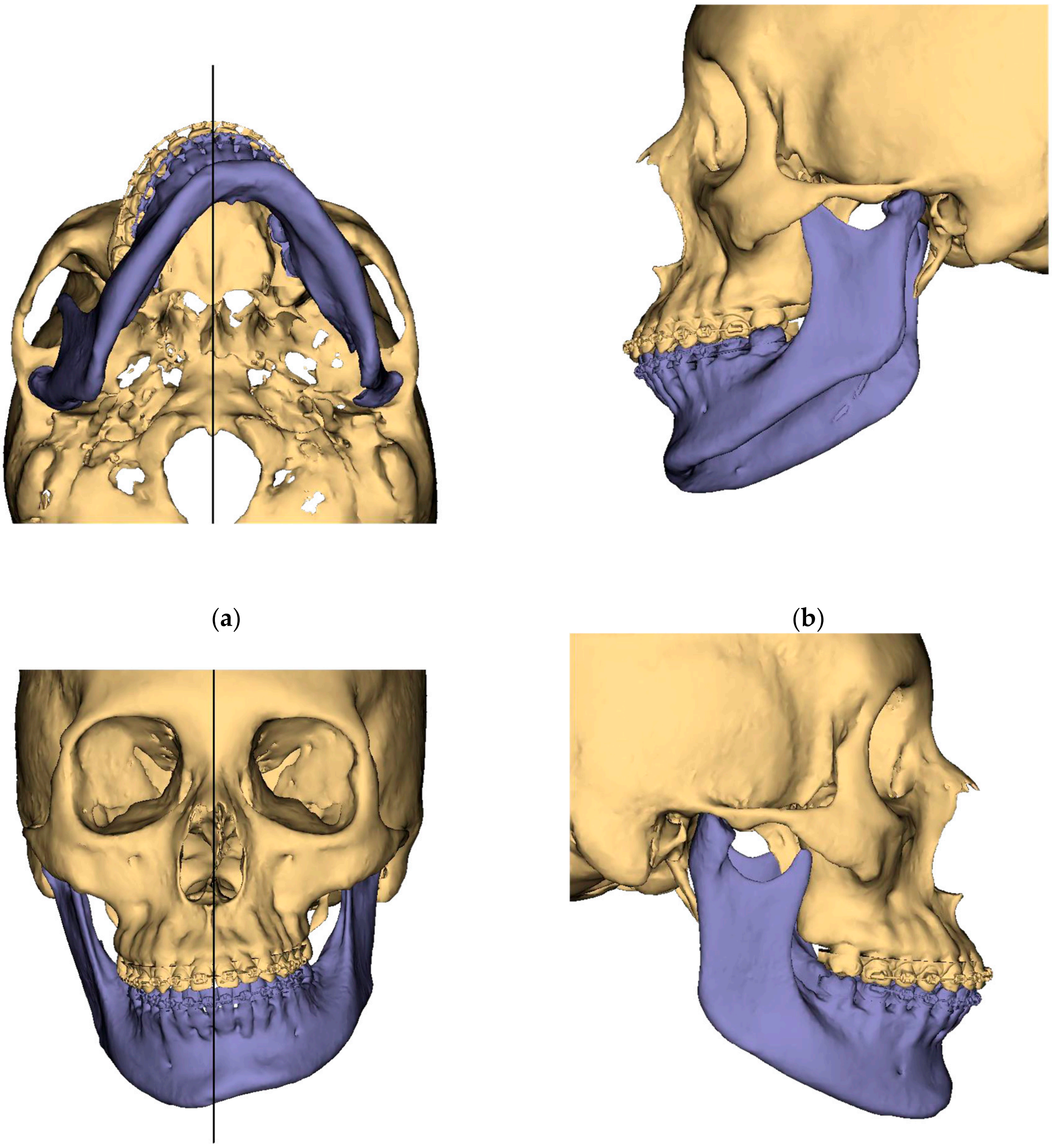

(**a**) (**b**)

(c) (d)

Figure S16. Surgical Plan: Pre-operative Position (**a**) Pre-operative situation of the patient showing asymmetry; (**b**,**c**,**d**) Pre-operative situation from lateral left (b), frontal (c) and lateral right sides.

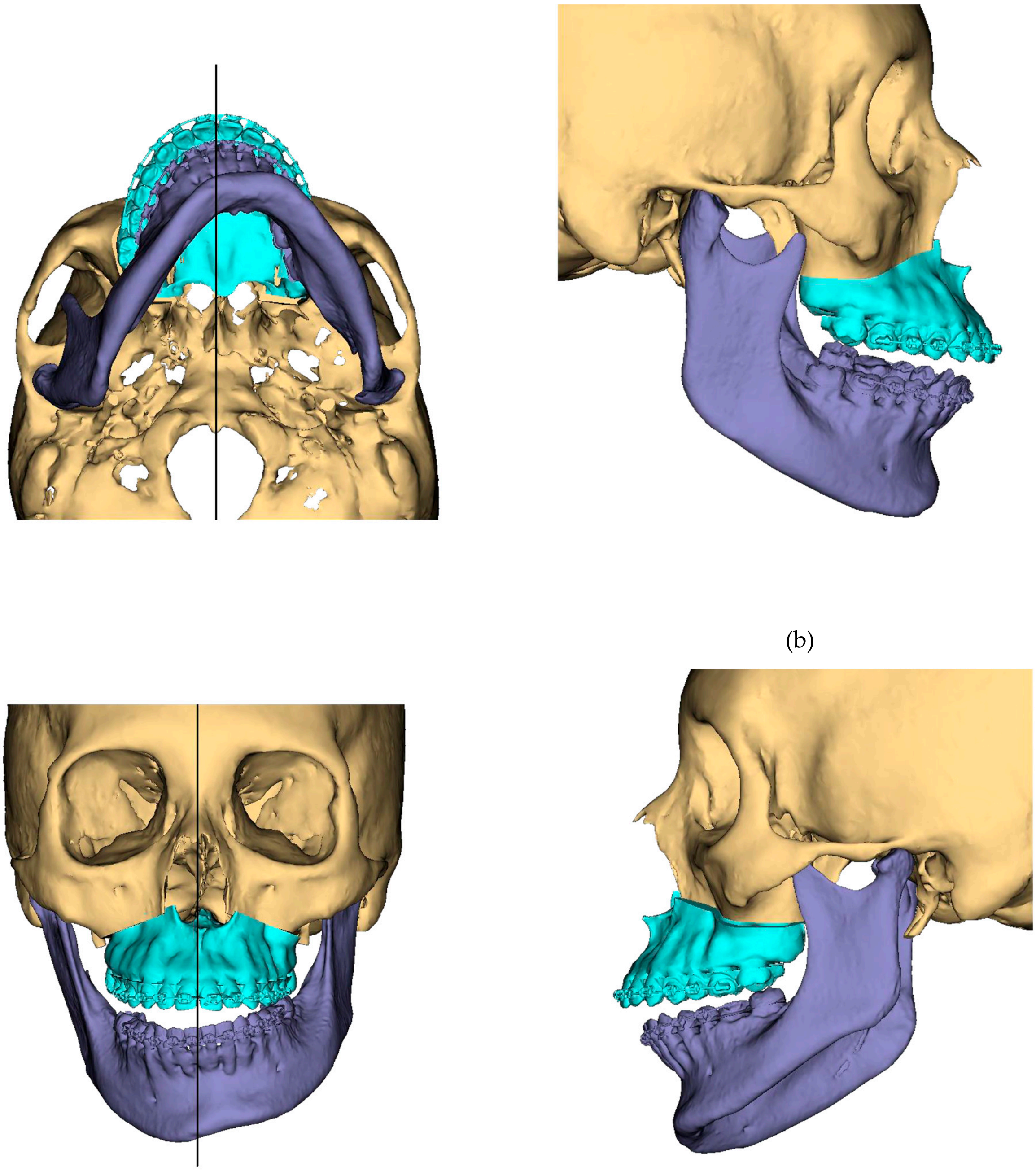

(a) (b)

(c) (d)

Figure S17. Surgical Plan: Intermediate Position (**a**) Planning for Maxillary movement first; (**b**,**c**,**d**) Pre-operative planning for maxillary movement from lateral left (b), frontal (c) and lateral right sides.

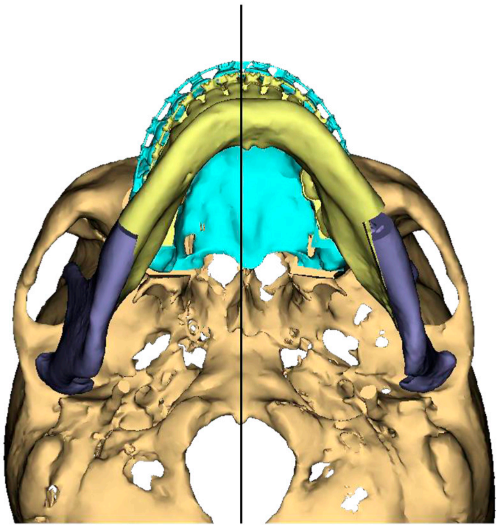

(**a**)

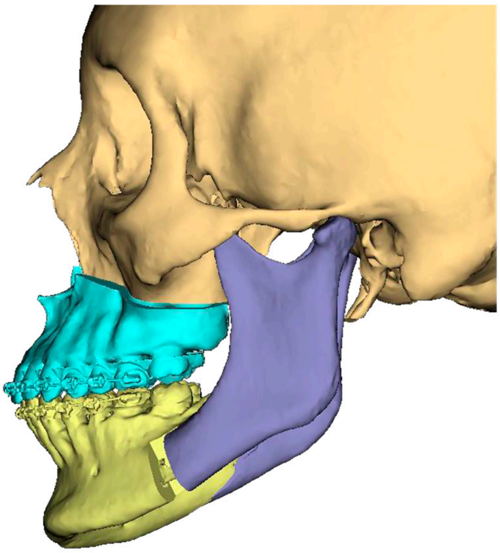

(**b**)

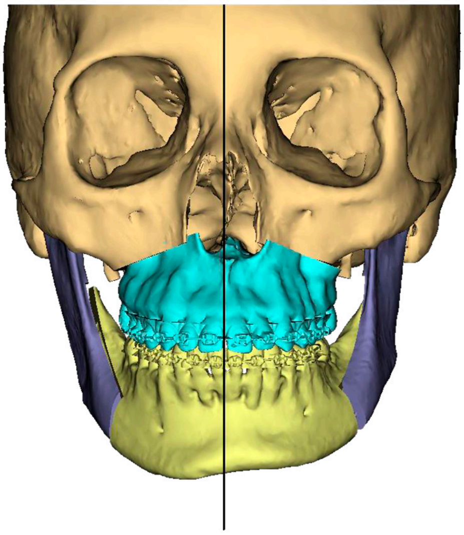

(c)

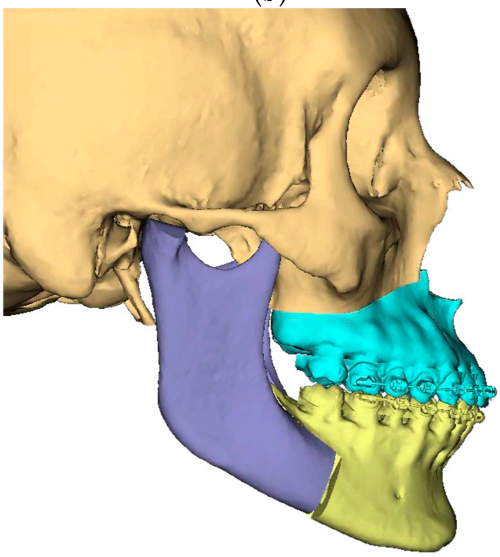

(d)

Figure S18. Surgical Plan: Final Position (**a**) Planning for mandibular position; (**b,c,d**) Pre-operative planning for sagittal-splint osteotomies of the mandibular bone maxillary movement from lateral left (b), frontal (c) and lateral right sides.

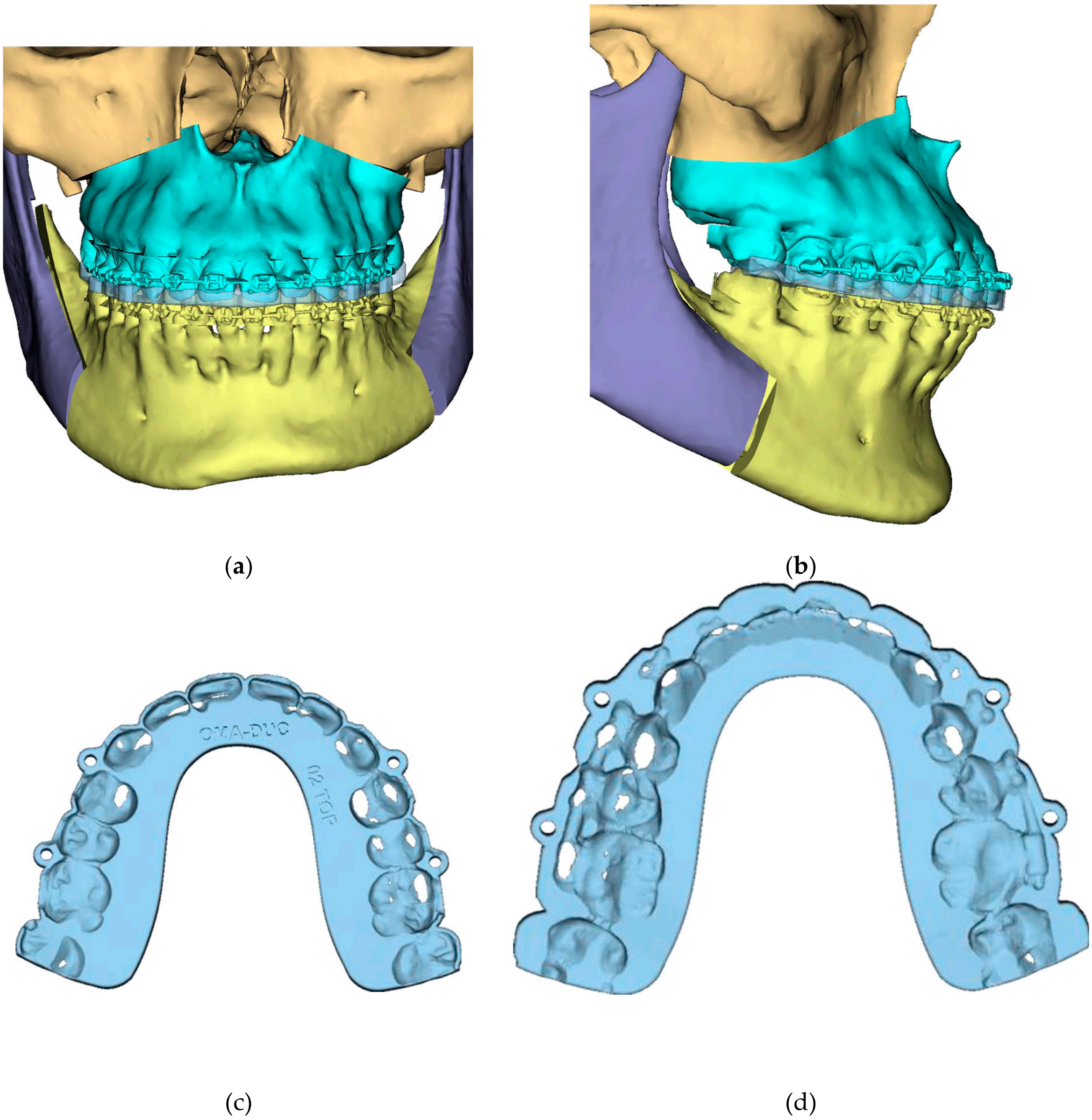

Figure S19. (a-d) Surgical Planning for Intermediate Splints that were used in Maxilla surgery first, that were used as guidance for bilateral mandular Sagital Split Osteotomy surgeries

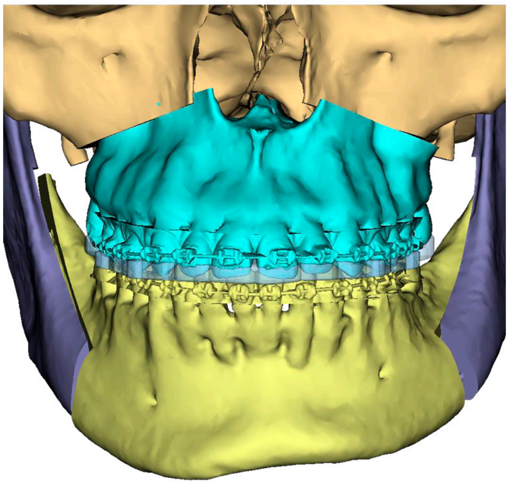

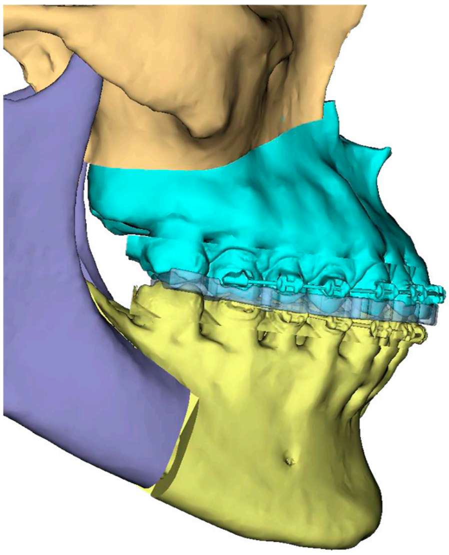

(a) (b)

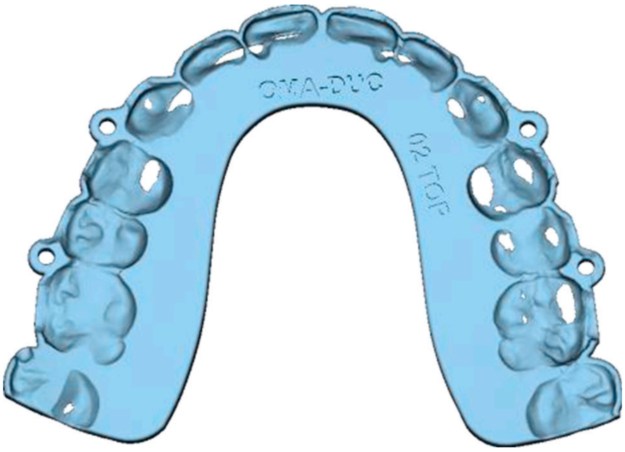

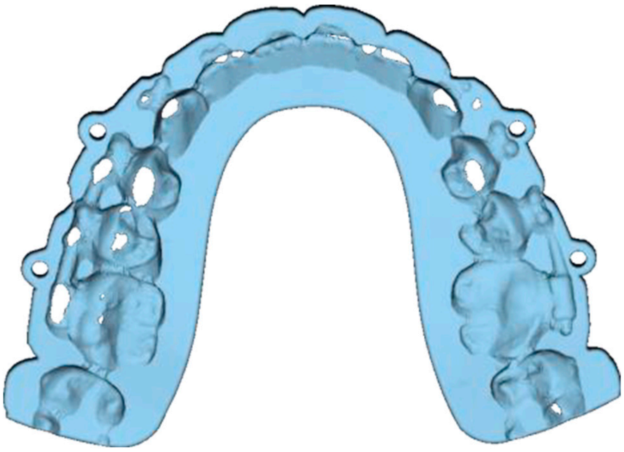

(c) (d)

Figure S20. (a-d) Surgical Planning for Final Splints that were used in Maxilla surgery first, that were used as guidance for bilateral mandular Sagital Split Osteotomy surgeries

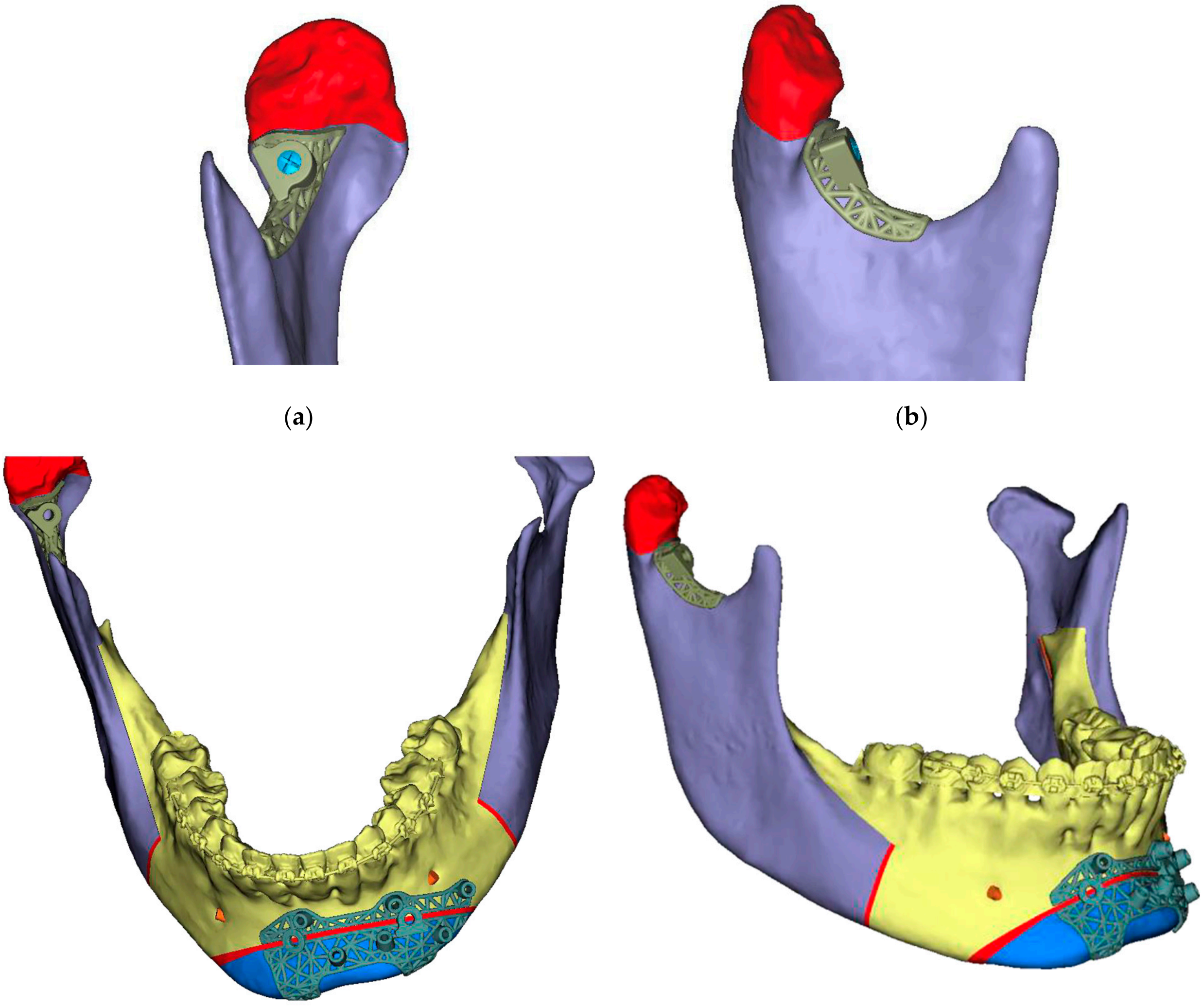

(a) (b) (c) (d)

Figure S21. (a-b) Titanium 3D printed guide for condylectomy from different angles, **(c-d)** Titanium 3D printed guide for genioplasty from different angles

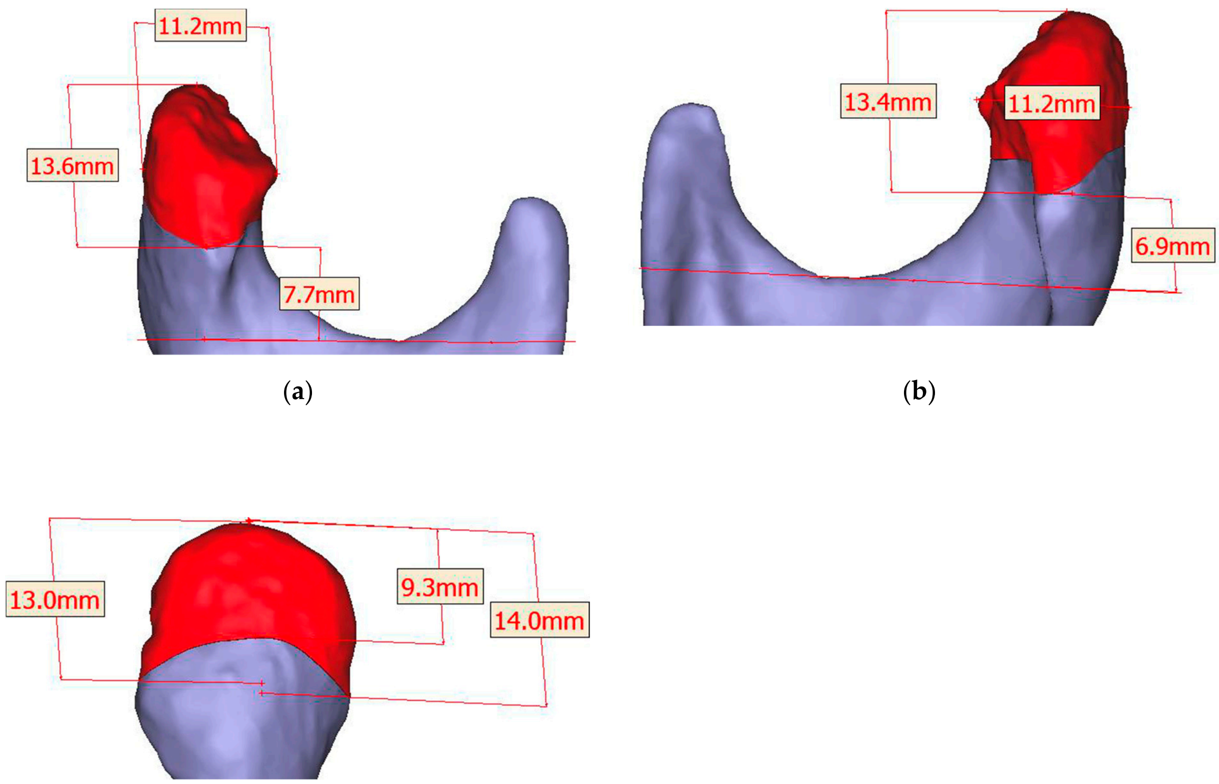

(**a**) (**b**)

(c)

Figure S22. (a-c) Planned Ramus trimming details

Surgical Plan: Cephalometric Analyses

Left: Cephalometric Analyses; Right: Overlay simulated planned soft tissue (light blue) with pre-operative soft tissue (dark blue)

Point	Left/Right (mm)		Ant/Post (mm)		Up/Down (mm)	
**A**	2.1	L	2.0	Ant	2.0	Up
**ANS**	2.8	L	2.0	Ant	2.1	Up
**Maxilla Incisor Midline**	0.3	L	2.0	Ant	2.0	Up
**Maxilla Canine L**	0.0	N/A	2.0	Ant	0.0	Down
**Maxilla Canine R**	0.2	R	2.0	Ant	3.8	Up
**Maxilla 1st Molar L**	0.4	L	2.0	Ant	0.9	Down
**Maxilla 1st Molar R**	0.1	L	2.0	Ant	4.7	Up
**B**	2.7	R	3.0	Ant	2.0	Up
**Pog**	9.2	R	3.7	Ant	0.1	Down
**Me**	10.1	R	4.0	Ant	0.6	Down
**Mandible Incisor Midline**	0.2	R	2.3	Ant	3.0	Up
**Mandible Canine L**	0.0	L	1.7	Ant	0.8	Up
**Mandible Canine R**	0.2	R	2.6	Ant	4.4	Up
**Mandible 1st Molar L**	0.5	R	1.3	Ant	1.6	Down
**Mandible 1st Molar R**	0.8	R	2.8	Ant	4.3	Up

Measurement (°)	Pre-Op 3D	Planned 3D	Delta 3D
**SNA**	88.0	90.3	2.3
**SNB**	82.6	84.6	2.0
**ANB**	7.0	5.8	-1.3
**Maxillary Occlusal Plane**	5.8	2.1	-3.6

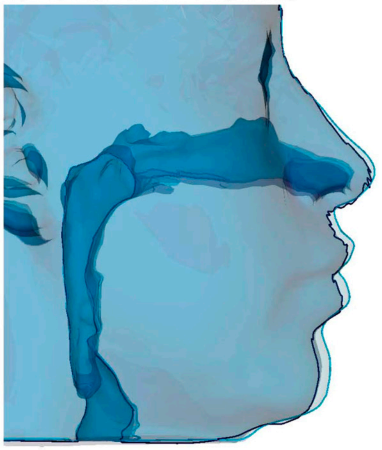

Note: A soft tissue simulation displays a prediction of the behavior of the patient's soft tissue aft surgically modifying the facial skeleton. Although the biomechanical algorithm has been successfu validated on real-life cases, no guarantees are given on the accuracy of the predicted outcome specific patients or surgical routines. Interpret the results with clinical judgement.

Kit reference		N/A
**Item reference**	**ID**	**Preview**
CMF.Ti074D	I1	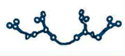
CMF.Ti054D	G1	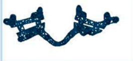
CMF.Ti070D	I2	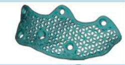
CMF.Ti053D	G2	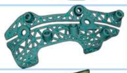
CMF.Ti053D	G3	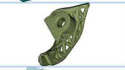
CMF.CA041	01	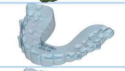
CMF.CA041	02	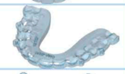
CMF.CA011	M1	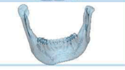

(**a**) (**b**)

Figure S23. (**a**) Cephalometric analyses (**b**) Delivered personalized medical devices for surgery (From up to down: Maxillary fixing plate, Maxillary cutting guide, genioplasty fixing guide, genioplasty fixing plate, Intermediate splint, Final splint, Mandibular model).

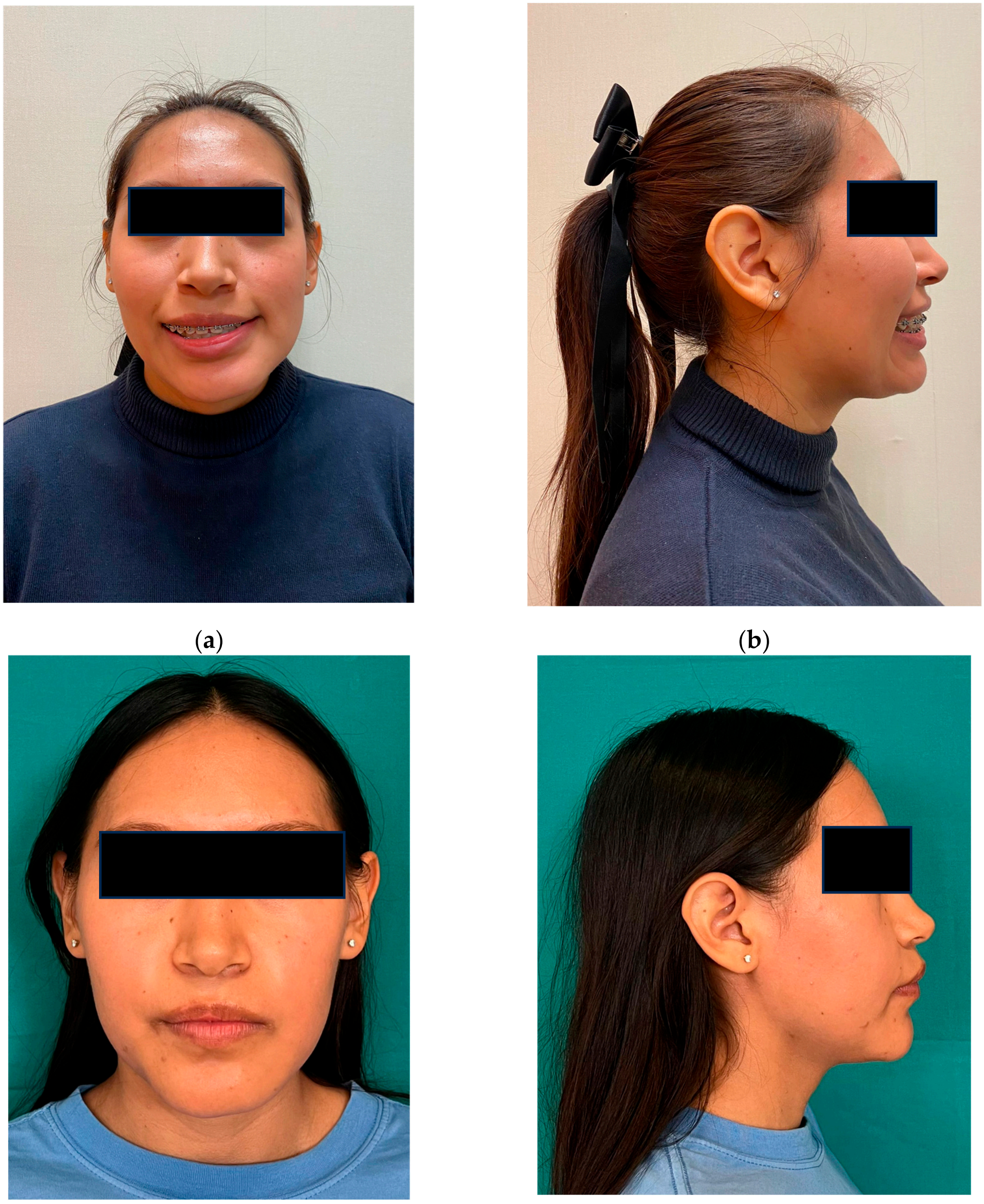

Figure S24: (a) Frontal and profile views showing pre-operative and **(b)** post-operative photographs of the same patient.

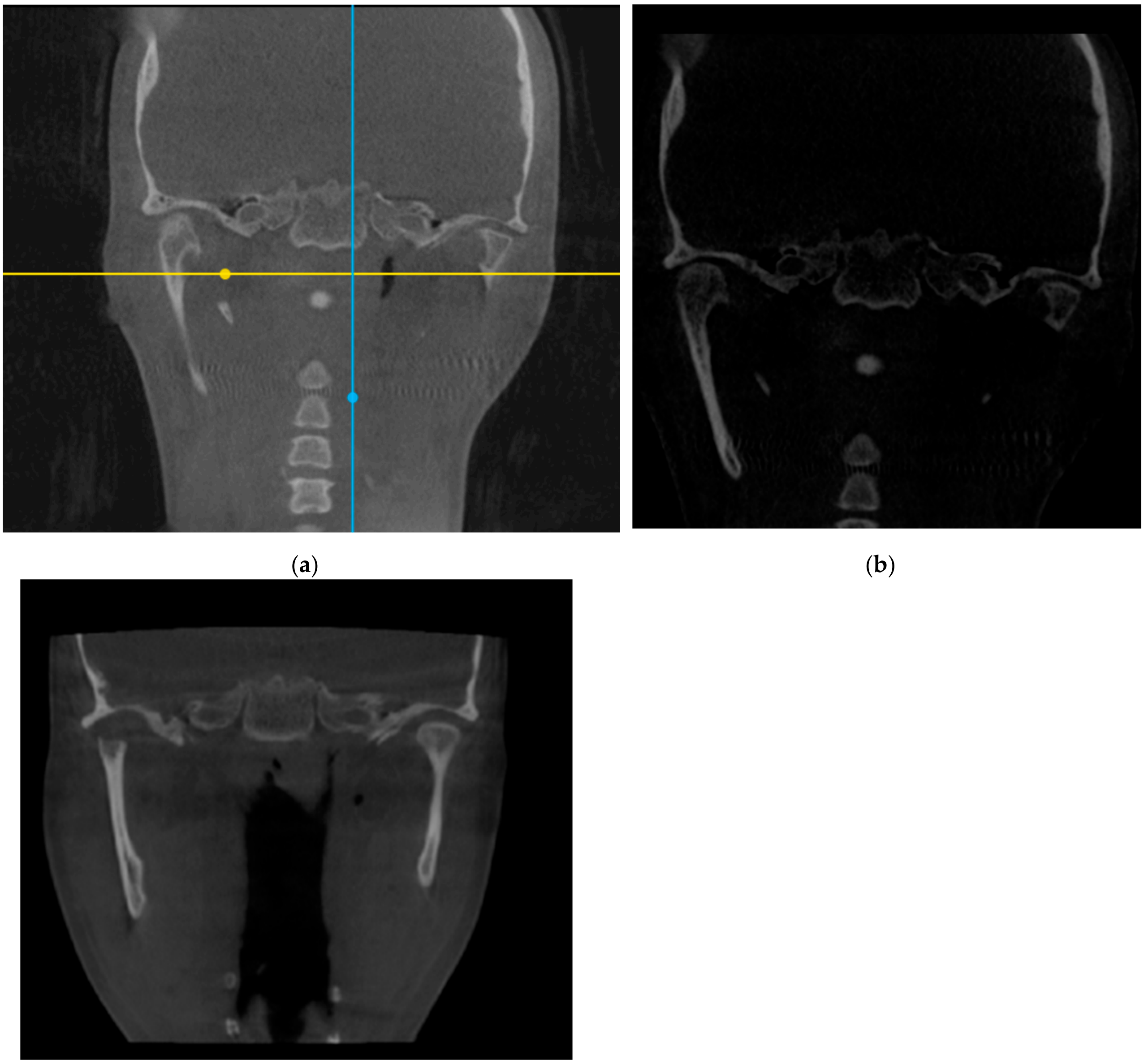

(**a**) (**b**)

(c)

Figure S25. (a-b) pre-operative and (**c)** Post- operative CBCT views of the same patient.
